# Cultivation‐Based Detection of a Novel High‐GC *Nitrospira* Derived From the Argentinian Copahue Volcano Area

**DOI:** 10.1111/1462-2920.70290

**Published:** 2026-04-03

**Authors:** Eva Spieck, Hanna Koch, Linnea F. M. Kop, Sabine Keuter, Marcel Malinowski, Katharina Sass, Wolfgang Sand, Edgardo Donati, Pablo Perez Garcia, Sebastian Lücker, Alejandra Giaveno

**Affiliations:** ^1^ Department of Microbiology and Biotechnology, Institute of Plant Sciences and Microbiology University of Hamburg Hamburg Germany; ^2^ Bioresources Unit, Center for Health & Bioresources, AIT Austrian Institute of Technology GmbH Tulln an der Donau Austria; ^3^ Department of Microbiology, Radboud Institute for Biological and Environmental Sciences Radboud University Nijmegen the Netherlands; ^4^ Division of Microbial Ecology, Centre for Microbiology and Environmental Systems Science University of Vienna Vienna Austria; ^5^ Institute of Carbon Cycles, Helmholtz‐Zentrum Hereon GmbH Geesthacht Germany; ^6^ Institute for General Microbiology Christian‐Albrechts‐Universität zu Kiel Kiel Germany; ^7^ Aquatic Biotechnology, Faculty of Chemistry University Duisburg‐Essen Essen Germany; ^8^ Institute for Oceanology of the Chinese Academy of Sciences in Qingdao Qingdao Shandong People's Republic of China; ^9^ CINDEFI (CCTLaPlata‐CONICET, UNLP), Facultad de Ciencias Exactas Universidad Nacional de La Plata La Plata Argentina; ^10^ PROBIEN (CONICET‐UNCo), Departamento de Química, Facultad de Ingeniería Universidad Nacional del Comahue Neuquén Argentina

**Keywords:** geothermal system, nitrification, nitrite‐oxidizing bacteria, *Nitrospira*

## Abstract

Nitrification is an essential process within the global nitrogen cycle and also occurs under extreme conditions, such as in geothermal environments. The nitrite‐oxidizing group *Nitrospira* represents key nitrifiers in these systems, as several species inhabit hot springs worldwide. Using different initial incubation temperatures, two novel moderately thermophilic *Nitrospira* enrichments, *Nitrospira* sp. Vd2 and *Ca*. N. *neuquenensis* E2OT, were obtained from sulfur‐rich mud pools in the geothermal field Las Máquinas (Neuquén Province, Argentina). *Nitrospira* sp. Vd2 belongs to the N. *bockiana* lineage V, whereas the second enrichment (E2OT) represents the novel taxonomic lineage VIII, together with cultures from Kamchatka (Kam‐Ns4a) and Garga hot springs (Ga3a). The vibrioid morphology of *Ca*. N. *neuquenensis* E2OT is strikingly different from all described, twisted rod‐shaped *Nitrospira*. Our study expands the knowledge of the taxonomic and genomic diversity of moderately thermophilic *Nitrospira*, by comparing the high‐quality draft genomes with those of previously described species. The recent discovery of quorum‐sensing genes outside the *Nitrospira* lineage II was confirmed for both Argentinian cultures. Notably, the genome GC contents of the enrichments Vd2 and E2OT are 60.6% and 69.4%, respectively. The latter is the highest observed for *Nitrospira* to date and might support thermotolerance up to 50°C.

## Graphical abstract

In this cultivation‐based study, two novel moderately thermophilic *Nitrospira* enrichments were obtained from the geothermal field Las Máquinas in the Neuquén Province, Argentina. Both nitrite oxidizers, belonging to lineage V or the novel taxonomic lineage VIII, contain quorum‐sensing genes and a high genome GC content of 60.6% and 69.4%.

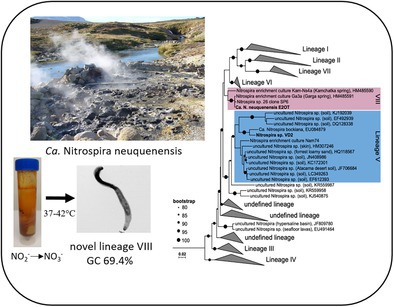

## 1 Introduction

Geothermal systems are found worldwide, and despite the high temperatures, various nitrogen (N)‐cycling processes are active (Wang et al. 2023). In these extreme environments, nitrifiers that oxidize ammonia to nitrite and further to nitrate are vital players within the microbial communities and in situ rate measurements in geothermal springs revealed that nitrification is functioning at temperatures above 80°C (Dodsworth et al. 2011; Reigstad et al. 2008). Nevertheless, thermophilic ammonia and nitrite oxidizers represent microorganisms that can only be enriched or isolated using specialized cultivation strategies (de la Torre et al. 2008; Hatzenpichler et al. 2008; Spieck, Spohn, et al. 2020; Spieck, Sass, et al. 2020).

The Copahue volcano geothermal area in Argentina (Neuquén province) is an extreme sulfur‐ and iron‐rich environment with a wide range of acidity and temperature conditions, leading to a high microbial diversity (Bedogni et al. 2019; Chiacchiarini et al. 2010; Urbieta, Porati, et al. 2015). So far, mainly thermophilic sulfur‐ or iron‐oxidizing bacteria and archaea have been isolated from this habitat, but also sulfate‐reducing microorganisms are present in anoxic hot spring sediments (Giaveno et al. 2013; Urbieta, Toril, et al. 2014; Willis et al. 2019). The area investigated here, Las Máquinas, is a pristine site featuring various geothermal ponds with moderate temperatures and acidic pH values (Urbieta, Porati, et al. 2015), where chemolithoautotrophic growth is widespread (Urbieta, Rascovan, et al. 2014). In addition to the detection of lineage II *Nitrospira* similar to N. *moscoviensis* (Urbieta, González‐Toril, et al. 2015), it represents a promising system for identifying and isolating novel nitrite‐oxidizing bacteria (NOB). Recently, the *Thermomicrobiota‐* (formerly *Chloroflexota*) affiliated bacterium *Nitrolancea copahuensis* was successfully isolated from this site (Spieck, Sass, et al. 2020), but other NOB have not yet been identified.

NOB are a phylogenetically heterogeneous bacterial group including members of the phyla *Pseudomonadota, Thermomicrobiota, Chloroflexota, Nitrospinota* and *Nitrospirota* (Daims et al. 2016). The most abundant and diverse genus of NOB is *Nitrospira* (Daims et al. 2001), which also includes complete ammonia‐oxidizing (comammox) species capable of performing both steps of nitrification (Daims et al. 2015; van Kessel et al. 2015). *Nitrospira* species are widely distributed across various natural environments, including soil (Nowka et al. 2015), freshwater (Podowski et al. 2022), the subsurface (Swanner and Templeton 2011), as well as marine and saline habitats (Daebeler et al. 2020; Mueller et al. 2023; Watson et al. 1986). In addition to natural habitats, *Nitrospira* are often found in high numbers in engineered systems, like communal wastewater treatment plants (WWTPs) (Daims et al. 2006; Spieck et al. 2006; Ushiki et al. 2013) and aquaculture biofilters (Foesel et al. 2008; Keuter et al. 2011; Kruse et al. 2013), and many isolates were obtained from these systems. Although their cultivation remains challenging, several representatives have been previously enriched or isolated from extreme geothermal settings, such as the Baikal rift zone and thermal springs (Edwards et al. 2013; Lebedeva et al. 2005). In general, *Nitrospira* are common members of the bacterial community of geothermal systems, especially at moderate temperatures below 50°C (Guo et al. 2020; Mitrović et al. 2022). In the biofilm of a thermal artesian spring with a water temperature of 63°C, a N. *calida* ‐like bacterium was even identified as the dominant community member (Marks et al. 2012). Moreover, a recent metagenomic study revealed the presence of thermophilic comammox *Nitrospira* in hot springs, alongside canonical NOB (Zhang et al. 2023); however, this habitat remains understudied (Palomo et al. 2022).

Phylogenetically, *Nitrospira* represents a deeply rooted group, whose taxonomic level is currently under debate (Kop et al. 2025). Genome‐based taxonomy (GTDB; Parks et al. 2018) splits the genus *Nitrospira* into several new genera (Glasl et al. 2024; Mueller et al. 2023), and cultivated *Nitrospira* are separated into 10 different genera across two families within the *Nitrospirales* (Kop et al. 2025). All described members of this order share the key metabolism of autotrophic nitrite oxidation and distinct morphological characteristics, such as their twisted‐rod shape and an extended periplasmic space, where the nitrite oxidoreductase (NXR) is localized (Spieck et al. 1996). The 16S rRNA gene‐based taxonomy of the genus *Nitrospira* divides it into eight distinct lineages (Daims et al. 2001; Kop et al. 2025). Of these, seven lineages are represented by cultured members and five lineages (II, V–VIII) include (moderately) thermophilic species. The first described member of lineage V—*Ca*. N. *bockiana*—originated from a steel pipeline of the Moscow heating system (Lebedeva et al. 2008). Representatives of lineage VI are N. *calida* , obtained from the Gorjachinsk hot spring in Russia, and the *Nitrospira* enrichment GaII from the Garga hot spring in Siberia. Another culture from the same sampling site (Ga3a), as well as the enrichment Kam‐Ns4a from a hot spring in the Uzon volcano caldera, have recently been assigned to the new lineage VIII (Kop et al. 2025). We changed the original name Ns4 (Lebedeva et al. 2011) to Kam‐Ns4a to avoid confusion with the *Nitrospirales* family NS‐4 classified via GTDB (Diamond et al. 2019; Rasmussen et al. 2025), in accordance with Kop et al. (2025). In addition, *N. tepida*, which was isolated from activated sludge using elevated temperature, forms the separate lineage VII (Keuter et al. 2023). Genome data from different *Nitrospira* species growing at high temperatures have only recently been published and compared (Keuter et al. 2023; Kop et al. 2025). However, *Nitrospira* bacteria from terrestrial thermal environments remain understudied despite their diversity.

Notably, a recent study investigated the survival mechanisms and habitat‐specific distribution of metagenome‐assembled genomes (MAGs) of comammox *Nitrospira* from hot springs (Zhang et al. 2023). The authors observed that the genomes of heat‐adapted species exhibit an elevated GC content (55.5%–59.3%). Supporting this finding, the moderately thermophilic species N. *moscoviensis* and *N. tepida* had the highest GC values of the described *Nitrospira* species (61.1%–62.0%) (Keuter et al. 2023; Koch et al. 2015). In contrast, mesophilic *Nitrospira* revealed lower GC values in the range of 48.5% to 59.9% (Bayer et al. 2021; Mueller et al. 2023).

In this cultivation‐based study, two novel *Nitrospira* enrichments were obtained using standard techniques, with an optical tweezer system employed for one of them. *Nitrospira* sp. Vd2, belonging to lineage V, is the third culture obtained from this lineage, alongside *Ca*. *N. bockiana* and *Nitrospira* sp. Nam74. *Ca*. N. *neuquenensis* E2OT is a novel species belonging to lineage VIII, represented so far only by two uncharacterized enrichment cultures (Kam‐Ns4a and Ga3a). It was investigated morphologically and physiologically with regard to temperature and the genomes of *Ca*. N. *neuquenensis* E2OT and *Nitrospira* sp. Vd2 were analysed in comparison to *Nitrospira* sp. Kam‐Ns4a and *Nitrospira* cultures belonging to lineages V, VI and VII.

## 2 Methods and Materials

### 2.1 Sampling

The acidic geothermal region of the Copahue volcano is located in the Neuquén province in Argentina (Figure S1; Urbieta, González‐Toril, et al. 2015). The area investigated here, Las Máquinas (Figure S2a), contains numerous thermal fluid emission systems, including fumaroles, boiling‐bubbling water and mud pools, with wide ranges of pH (2–7) and temperatures (30°C–90°C) (Willis Poratti et al. 2016). Sediment slurry and water of two sulfur‐rich mud pools in Las Máquinas were sampled in December 2008 (E2, source of E2OT) and in March 2011 (A4, source of Vd2; Figure S2b,c). Mud sample E2 formed a red biofilm with chemical precipitation on the rim of the sampling tube, whereas the water sample A4 contained reddish brown flocs (Figure S3). The sampling site of E2 had a water temperature of 40°C, a pH of 2.5–3.0 and an ammonium concentration of 10 mg l^−1^. Site A4 had a temperature of 45°C, a pH of 6.0 and ammonium was below the detection limit. Water chemistry values were published previously (Spieck, Sass, et al. 2020).

### 2.2 Cultivation

*Nitrospira* was enriched in 300 mL Erlenmeyer flasks as batch cultures in a mineral NOB salts medium described previously (Spieck and Lipski 2011), using 0.3–5 mM NaNO_2_ as substrate. From 2018 on, 1 mL l^−1^ of trace elements according to Widdel and Bak (1992) instead of those in the original salts medium were added to the medium, containing 100 mg l^−1^ MnCl_2_ × 4 H_2_O, 30 mg l^−1^ H_3_BO_3_, 144 mg l^−1^ ZnSO_4_ × 7 H_2_O, 36 mg l^−1^ Na_2_MoO_4_ × 2 H_2_O, 2.1 g l^−1^ FeSO_4_ × 7 H_2_O, 2 mg l^−1^ CuCl_2_ × 2 H_2_O, 190 mg l^−1^ CoCl_2_ × 6 H_2_O and 24 mg l^−1^ NiCl_2_ × 6 H_2_O.

About 150 mL of medium was inoculated with 1 mL of the sample containing sediment material and/or water. Cultures were grown under static conditions, with the substrate repeatedly replenished when consumed (0.25–2.5 mM NaNO_2_). When cultures were fed about 6–10× and had consumed at least 3 mM of substrate, cells were transferred in 1% (v/v) inoculum to fresh mineral NOB media. From the sample material of E2, a culture was initiated at 28°C shortly after sampling in 2008, changed to 34°C in 2011, and after cell sorting at 37°C–42°C partly with shaking at 125 rpm (E2OT; Figure S3). For the cultures grown at 42°C (Vd2), the sample material from both sites was stored at room temperature in the dark for 2 years (sample E2) or 4 years (sample A4) before inoculation into Erlenmeyer flasks. In 2023, a new subculture of sample A4 incubated at 50°C was initiated by inoculating it with 1% (v/v) of the nitrite‐oxidizing culture enriched at 42°C (Figure S3). Biomass of the E2OT culture grown with 5 mM NaNO_2_ at 42°C and the Vd2 culture grown with 3 mM NaNO_2_ at 37°C, sampled in 2008 or 2011, was used for metagenomic sequencing (see below).

### 2.3 Physiological Experiments

Nitrite consumption was used as an indication of growth and determined using the Griess‐Ilosvay spot test (Schmidt and Belser 1994). The optimal growth temperature was investigated in batch cultures using 50 mL of mineral medium in 100 mL Erlenmeyer flasks and with 1.4 mM nitrite at temperatures ranging from 17°C to 55°C. Medium was inoculated with 1% (v/v) of active precultures grown at 28°C or 37°C, and the test was conducted in one (culture E2) and three biological replicates (enrichment E2OT). Nitrifying activity was analysed quantitatively via HPLC for 27 or 14 days. Afterwards, nitrite oxidation at suboptimal temperature was further monitored qualitatively by the Griess–Ilosvay spot test. The temperature optimum for Vd2 was investigated in the range of 17°C to 50°C, using 0.5 mM nitrite, in triplicate glass tubes containing 10 mL of medium inoculated with 0.2 mL of preculture. The identity of *Nitrospira* in the enrichment cultures was checked repeatedly by 16S rRNA gene amplicon sequencing and 16S rRNA gene (primers 27f/1158r; Lane 1991; Maixner et al. 2006) and *nxrB* PCR (primers 169f/638r and 19f/1237r; Pester et al. 2014) in combination with cloning and Sanger sequencing.

### 2.4 Chemical Analyses

Nitrite and nitrate concentrations were determined quantitatively by HPLC via ion pair chromatography on a LiChrospher RP‐18 column (125 × 4 mm; Merck) with UV detection in an automated system (Hitachi LaChrom Elite; VWR International GmbH, Darmstadt, Germany). Data acquisition and processing of nitrite and nitrate concentrations were performed with the integrated software EZChrom Elite 3.3.2.

### 2.5 Isolation Attempts

After 8 months of growth at 34°C, cells from the enrichment culture of sample E2 were manually separated by an optical tweezer system (PALM MicroTweezers, Carl Zeiss Microscopy GmbH, Munich, Germany) as described in Nowka et al. (2015). Single cells of interest were trapped and separated with an infrared laser (1064 nm, 3 W) through a 30 μL volume capillary. Laser trapped cells, still accompanied by a few floating cells, were transferred to an Eppendorf tube containing 1.5 mL NOB medium with 0.3 mM NaNO_2_ and active nitrite oxidizing cultures were gradually transferred to larger cultures vessels. After growth of recovered cells was established in Erlenmeyer flasks, the culture was filtered twice using 0.45 μm filters and incubated with fresh medium, partly shaken at 37°C and supplied with up to 5 mM substrate. Highly enriched cultures were subjected to serial dilution in mineral medium containing 0.5 mM of nitrite. Culture purity was tested on solid RT (2.5 g l^−1^ meat extract, 2.5 g l^−1^ casamino acids, 0.5 g l^−1^ yeast extract, 1.0 g l^−1^ KH_2_PO_4_, 0.5 g l^−1^ NaCl, pH 7.4, 15.0 g l^−1^ agar in distilled water), R2A, or R2A half‐strength plates (Spieck, Spohn, et al. 2020).

### 2.6 Microscopy

For transmission electron microscopic observations, cells were collected by centrifugation (13,000×*g*, 15 min), fixed and embedded as described by Spieck and Lipski (2011). Ultrathin sections, as well as cells stained with uranyl acetate for whole cell morphology (Spieck et al. 1996), were analysed using a transmission electron microscope (Zeiss model Leo 906E with a CCD camera model 794; Carl Zeiss, Jena, Germany).

Light microscopic images were taken with an AxioScope epifluorescence microscope equipped with a N‐Achroplan 100× 1.25 oil objective and an AxioCam ICc1 1.4‐megapixel CCD camera (Zeiss, Jena, Germany).

### 2.7 Fluorescence In Situ Hybridization

Cells were pelleted at 10°C and 13,000×*g* for 15 min, washed with 0.9% (w/v) NaCl and fixed with 3% PFA as described previously (Amann et al. 1995). After dehydration in 50%, 80% and 96% ethanol for 3 min each (Manz et al. 1992), cells were hybridized in hybridization buffer containing 35% formamide with the FITC‐labelled universal bacterial probes EUBI‐III 338 (Amann et al. 1990; Daims et al. 1999) and the Cy3‐labelled probe S‐G‐Ntspa‐0662‐a‐A‐18 specific for the genus *Nitrospira* together with the competitor probe (Daims et al. 2001). Following hybridization, samples were stained with 4′,6′‐diamidino‐2‐phenylindole (DAPI) and embedded in Citifluor AF1 (Citifluor Ltd., London, United Kingdom). Labelled cells were visualized using an LSM 800 confocal laser scanning microscope with an Airyscan (Zeiss, Jena, Germany) equipped with Plan‐Apochromat 63× and 100× 1.4 oil objectives.

### 2.8 DNA Extraction

For DNA isolation, biomass was harvested via centrifugation (13,000×*g* for 15 min at 10°C). For 16S rRNA gene amplicon and metagenome sequencing of the enrichment Vd2, the PowerSoil DNA isolation kit (MO BIO Laboratories, USA) was used according to the manufacturer's instructions with an additional enzyme treatment (Proteinase K, lysozyme, RNase) according to Spieck, Sass, et al. (2020). For 16S rRNA gene amplicon and metagenome sequencing of E2OT and PCR‐based *nxrB* amplification of the E2 cultures, DNA was isolated with the PowerSoil DNA isolation kit without modifications. DNA isolation for 16S rRNA gene amplicon sequencing and *nxrB* PCR‐based amplification of the Vd2 culture was performed using the Bacterial & Yeast Genomic DNA Kit from Roboklon (Germany), following the manufacturer's instructions for bacteria. DNA quality and concentrations for all samples were determined using a NanoDrop 2000 spectrophotometer (Thermo Fisher Scientific, Waltham, MA, USA).

### 2.9 Amplicon Sequencing

16S rRNA gene amplification of *Ca*. N. *neuquenensis* E2OT and *Nitrospira* sp. Vd2 at different enrichment steps was performed using barcoded, V3/V4 region‐specific PCR primers (for details see supplemental material). OTU representative sequences and taxonomic assignments are listed in Table S1.

### 2.10 Metagenomic Sequencing and Assembly

Metagenome sequencing of the enrichment culture E2OT was performed by Eurofins (Ebersberg, Germany) using Illumina sequencing, generating 10 million paired‐end reads with a length of 2 × 150 bp (20 M reads in total). For metagenomic sequencing of the enrichment Vd2, 1 ng of DNA was used for tagmentation and sequencing libraries were prepared using the Nextera XT kit (Illumina, San Diego, CA, USA) according to the manufacturer's protocol. The quality of all MAGs obtained from the two metagenomes was assessed using checkM2 v1.0.1 (Chklovski et al. 2023). MAGs were taxonomically classified using GTDB‐Tk (v2.4.0) (Chaumeil et al. 2022) and the GTDB database R226. The mean coverage of all MAGs was calculated using coverM v0.7.0 (Aroney et al. 2025) and the Illumina raw reads of the metagenomes. Selected genes were extracted from the metagenome assemblies with a minimum contig length of 1000 bp using anvi'o v.8 (Eren et al. 2021). For 16S rRNA genes, anvi‐run‐hmms and anvi‐get‐sequences‐for‐hmm‐hits—hmm‐source Ribosomal_RNA_16S were employed. For Vd2, all hits of COG0243 were identified using the COG20 database, extracted and surveyed via blastp, which identified one *Nitrospira* NxrA. The NxrAB and 16S rRNA gene sequences can be obtained from https://doi.org/10.5281/zenodo.17235877. A detailed description of the *Nitrospira* genomes analysis including average nucleotide identity (ANI) and phylogenetic analyses is available in Supporting Information.

## 3 Results

### 3.1 Enrichment of Novel *Nitrospira*

From the thermal area Las Máquinas (Figure S1), two new representatives of *Nitrospira* were highly enriched from a mud and a water sample (E2 and A4, respectively; Figure S2) using varying incubation temperatures ranging from 28°C to 50°C (Figure S3). The *Nitrospira* culture Vd2, affiliated with *Ca*. *N. bockiana* lineage V, was enriched at 42°C from both samples in mineral NOB medium without further supplements, providing nitrite as substrate and as sole N source. In addition, the *Nitrospira* enrichment E2OT (tentatively named *Ca*. N. *neuquenensis* ) could be selectively enriched using the mud sample E2 as inoculum and 28°C as starting incubation temperature. The same *Nitrospira* was enriched when sample A4 was cultivated at 50°C. In the following paragraphs, the monitoring of the enrichment process for the separate cultures (Vd2 and E2OT) is described in more detail. Despite repeated attempts to obtain pure cultures, both enrichments still contain heterotrophic bacteria.

### 3.2 Enrichment of *Nitrospira* sp. Vd2

After storage of the mud sample E2 at room temperature for 27 months, NOB were enriched in standard mineral NOB medium at 42°C. According to 16S rRNA gene amplicon sequencing (Figure S4), the culture contained 3.8% *Nitrospira* sp. Vd2 after 4 years of incubation at 42°C and was further enriched to 40% after 5 years of incubation.

Inoculation of NOB medium with water sample A4 in 2015, and incubation at 42°C for 6 years resulted in the enrichment of *Nitrospira* sp. Vd2 to 61% relative abundance.

### 3.3 Enrichment of *Ca. Nitrospira neuquenensis* E2OT

**FIGURE 1:**
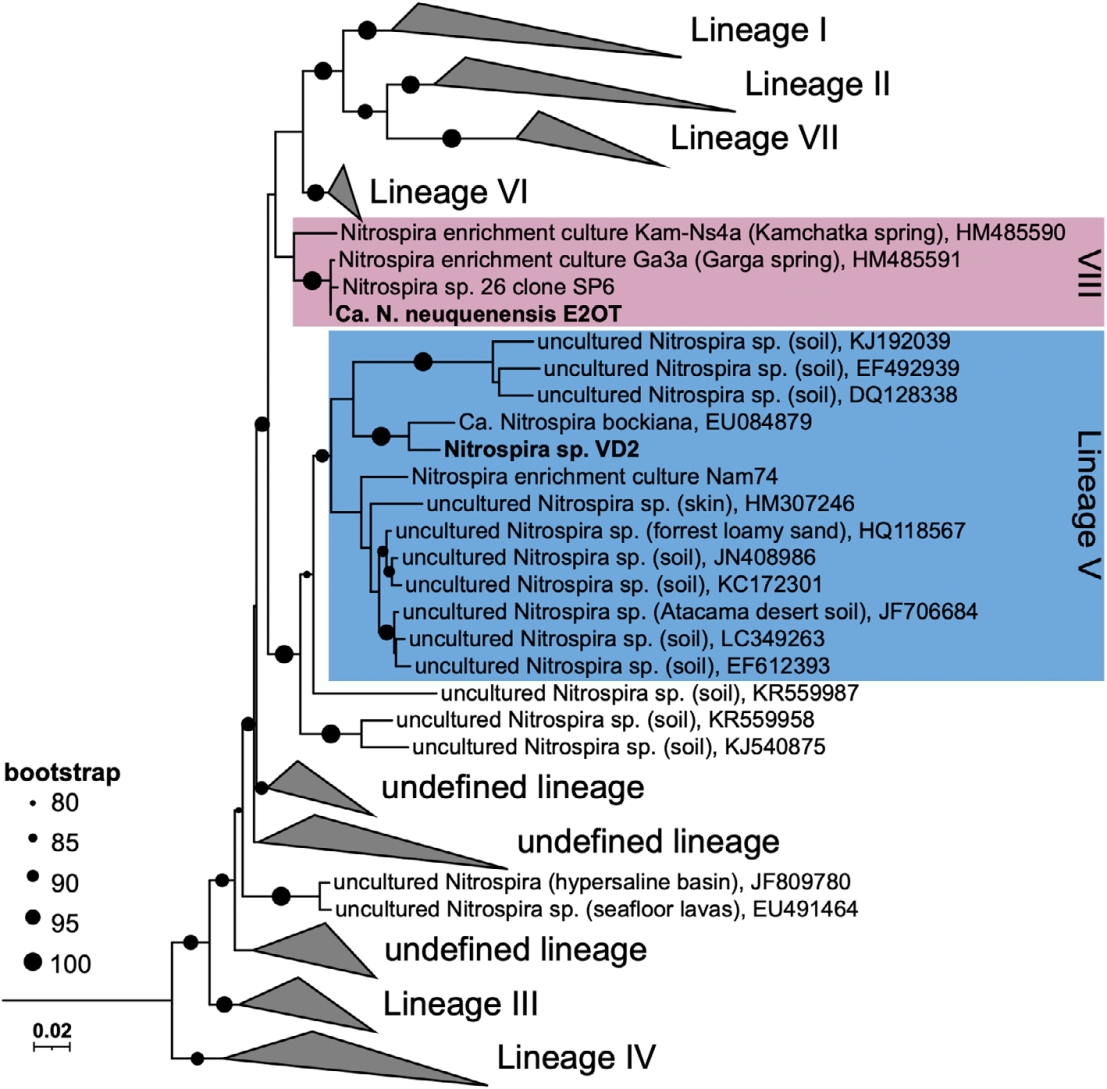
Phylogenetic affiliation of *Ca. Nitrospira neuquenensis* E2OT and *Nitrospira* sp. Vd2 based on 16S rRNA gene sequences. The coloured areas in blue mark lineage V (*Ca. N. bockiana*‐like sequences), in red the new lineage VIII (E2OT). *Ca*. N. *neuquenensis* E2OT and *Nitrospira* sp. Vd2 are labelled in bold. Circles indicate bootstrap support between 80% and 100% of 1000 ultrafast bootstrap replications. Five *Thermodesulfovibrio* and two *Leptospirillium* sequences were used as outgroup to root the tree. For details see Table S2.

An incubation of sample material from E2 was started at 28°C, but the optimal growth temperature of the present NOB was determined to be 34°C (Figure S5). Therefore, the incubation temperature was increased to 34°C 2 years after initial enrichment of NOB. Here, a previously unknown *Nitrospira* was identified by cloning and sequencing of the *Nitrospira nxrB*, and its 16S rRNA gene sequence (clone 26_SP6) was nearly identical to that of the later identified species *Ca*. N. *neuquenensis* E2OT (Figure 1). Separation of E2OT from some accompanying heterotrophic bacteria, as well as from other *Nitrospira*, was achieved by trapping apparent single cells with the laser of the optical tweezer and transferring them to culture medium in an Eppendorf tube for subsequent enrichment at 37°C. However, heterotrophic bacteria were still present as well as another *Nitrospira* closely related to N. *japonica* (98.2% 16S rRNA gene identity). The microcolony‐forming N. *japonica* ‐like bacterium was successfully removed by filtering twice through a 0.45 μm filter and the following cultures grown at 37°C–42°C contained only the *Nitrospira* representative E2OT. It was enriched to approximately 18% relative abundance after 1 year of cultivation based on 16S rRNA gene clone libraries (not shown). Finally, after 14 years, *Nitrospira* E2OT revealed a high degree of purity (Figures S6 and S7), and this new *Nitrospira* sp. was provisionally named *Ca*. N. *neuquenensis*.

Interestingly, *Ca*. N. *neuquenensis* E2OT was the only known NOB in nitrite‐oxidizing incubations at 50°C that were inoculated with enrichment cultures of the mud sample A4 grown at 42°C. When grown at 50°C, 16S rRNA gene amplicon sequencing revealed a 57% relative abundance of *Ca*. N. *neuquenensis* E2OT as sole NOB (Figure S7) and accordingly, a high cell number of *Nitrospira* was observed using FISH (Figure S8). Further physical separation via serial dilutions removed some accompanying bacteria, but no isolate is available up to now. Finally, biomass grown from the dilution step 10^−3^ at 50°C revealed a relative abundance of 93% for *Nitrospira* E2OT, 6.6% *Hyphomicrobium* and 0.2% *Paludibaculum* (Figure S7, Table S1).

### 3.4 Morphology and Physiology

**FIGURE 2:**
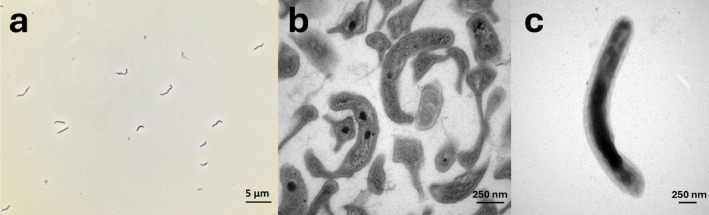
Cell morphology and ultrastructure of the new *Ca*. N. *neuquenensis* E2OT revealing the unusual cell shape. (a) Phase contrast of “S‐forms” consisting of several curved rods. The batch culture was grown at 37°C with 5 mM nitrite, (b) ultrathin section of long curved rods, large dark spots are considered to represent polyphosphate. The culture was incubated with 3 mM nitrite and extensively fed, incubation at 37°C, (c) negative staining of a long curved rod. The culture was grown at 42°C with 5 mM nitrite and used for genome sequencing.

**FIGURE 3:**
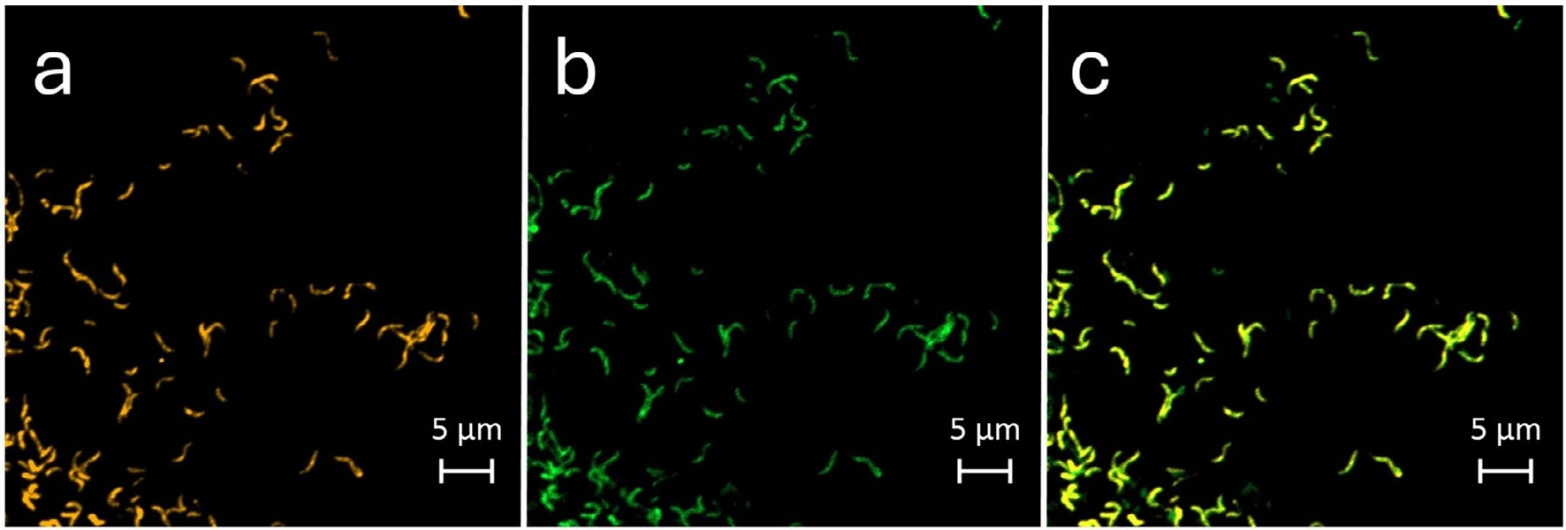
Fluorescence in situ hybridization of *Ca*. N. *neuquenensis* E2OT showing “S‐forms”. The culture was grown at 45°C without shaking and regularly fed with 0.5 mM for 19 months. (a) Genus‐specific probe Ntspa662, cy3, (b) EUB I‐III, FITC, (c) overlay.

Unlike all known cultivated *Nitrospira*, most *Ca*. N. *neuquenensis* E2OT cells appeared as curved rods that were single‐planed without clear twists with increasing cell length upon nitrite consumption (Figures 2 and S9). Single cells in the form of short vibrios with a size of 0.35–0.55 μm × 1–2 μm dominated in fresh cultures started with a low nitrite concentration (0.5 mM), especially under shaking (Figures S6, S8 and S9a). Using higher substrate concentrations of 3–5 mM in the beginning or after prolonged and intensive feeding regimes increased appearance of “S‐forms” due to incomplete cell division. These cells could extend to long snake‐like rods with up to 8.4 μm in length (Figures 2 and 3 and Figure S9b). A few flagella‐containing cells could be observed, indicating motility (Figure S9c). In ultrathin sections, cells appeared pleomorphic and the large periplasmic space typical for *Nitrospira* became visible together with storage material that most probably consists of glycogen and polyphosphate (Figure 2b; Bock 1976). Even after long‐term cultivation, no microcolonies were present, neither in static nor in shaken incubations (Figure S6) and biofilm formation was not observed.

In contrast, *Nitrospira* sp. Vd2 revealed the typical cell shape of this genus, showing about 2 μm long twisted rods (Figure S10a) and forming large microcolonies (Figures S10b and S11).

Reproducible growth of *Ca*. N. *neuquenensis* E2OT obtained from sample E2 was observed between 28°C and 45°C with an optimum at 42°C (Figure S12a). The culture consumed 1.4 mM nitrite within 12 days at 42°C (Figure S12b), whereas growth was delayed at 22°C and almost ceased at 50°C. Notably, reproducible growth of *Ca*. N. *neuquenensis* E2OT at 50°C only occurred with follow‐up cultures obtained from sample A4, which were not incubated at 28°C initially and were not subjected to physical cell separation. Alternatively, cultures derived from sample E2 became also active at 50°C, when vitamin B12 was added (0.02 μg/mL). The temperature range of *Nitrospira* sp. Vd2 was 22°C to 45°C, and best growth occurred at 33°C to 42°C (not shown).

### 3.5 Phylogeny of New *Nitrospira* Enrichments

**FIGURE 4:**
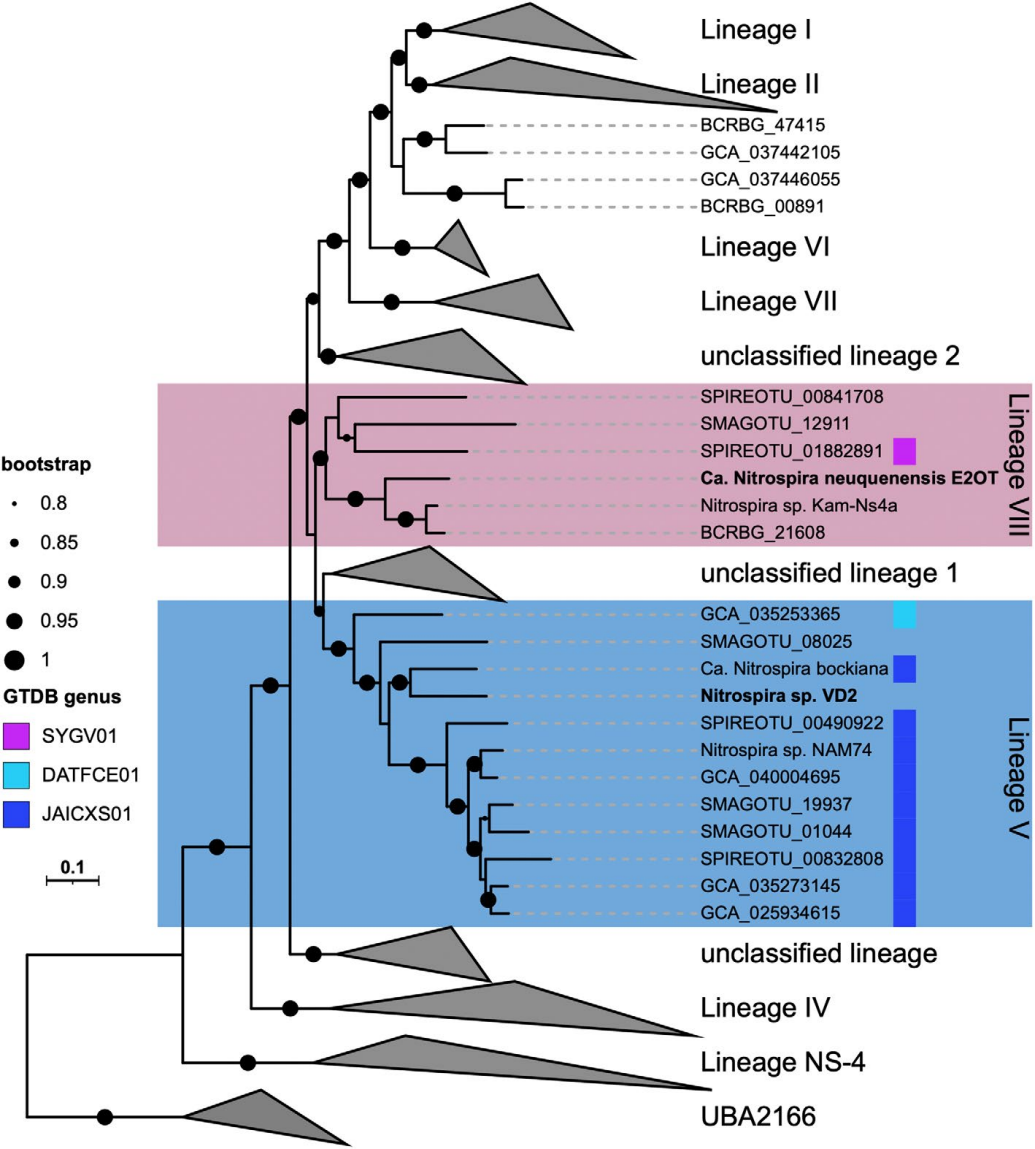
Phylogenomic classification of Ca. N. *neuquenensis* E2OT and *Nitrospira* sp. Vd2 based on the concatenated alignment of 71 conserved bacterial marker proteins. The genomes of *Ca*. N. *neuquenensis* E2OT and *Nitrospira* sp. Vd2 are labelled in bold. Names of *Nitrospirales* genomes included in GlobDB were omitted. Circles indicate Shimodaira‐Hasegawa test support between 80% and 100% of 1000 bootstrap replications. GTDB genera were identifed based on R226. For details see Figure S13 and Table S2.

Phylogenetic analyses based on 16S rRNA genes (Figure 1, Table S2) and the concatenated alignment of 71 conserved marker proteins (Figures 4 and S13) showed that *Ca*. N. *neuquenensis* groups together with the enrichment *Nitrospira* sp. Kam‐Ns4a within the novel *Nitrospira* lineage VIII recently delineated by Kop and colleagues (Kop et al. 2025). Lineage VIII forms a monophyletic group clearly separated from other described lineages. Notably, the environmental MAG (BCRBG_21608) that clusters to the thermophilic *Nitrospira* cultures was obtained by the coassembly of two metagenomes from hot springs across the Costa Rican convergent margin (Aroney et al. 2025; Rogers et al. 2023). The low ANI/AAI values (< 80%; Figure S14) of *Nitrospira* sp. Kam‐Ns4a and *Ca*. N. *neuquenensis* indicate a distant relationship within this cluster. Other environmental MAGs belonging to this lineage originated from tap water, subsurface soil and a WWTP (Figure 4, Table S3; Ng et al. 2019). However, their exact taxonomic affiliation based on GTDB is unclear since all lineage VIII MAGs except for one could not be assigned to a genus (Figure 4).

16S rRNA gene surveys using nBLAST against the nr nucleotide database revealed only a low similarity of *Ca*. N. *neuquenensis* E2OT to previously described *Nitrospira* species. Based on the 16S rRNA gene phylogeny, *Ca*. N. *neuquenensis* E2OT clusters together with the *Nitrospira* enrichments Ga3a and Kam‐Ns4a (98.2% and 96.1% identity), which originate from geothermal springs in the Baikal and Kamchatka regions, respectively (Lebedeva et al. 2011). The most closely related taxonomically described species is the lineage VI‐affiliated N. *calida* , which shares 95% gene sequence identity. The 16S rRNA gene sequence of *Nitrospira* sp. Vd2 is closely related to *Ca. N. bockiana* (97.9%) and clusters within lineage V together with sequences mainly detected in different soils (Figure 1). Concordantly, the genome of *Nitrospira* sp. Vd2 clusters within lineage V to other (moderately) thermophilic *Nitrospira*, namely *Ca. N. bockiana* (Figure 4) and *Nitrospira* sp. Nam74 (Kop et al. 2025). Lineage V includes the GTDB genera JAICXS01 and DATFCE01. The lineage V MAGs originated from soil samples from Antarctica, China, and the USA (Table S3; Ma, Lu, et al. 2023; Nayfach et al. 2021; Schmidt et al. 2024).

In addition, the nitrite oxidoreductase alpha (*nxrA*) and beta subunit (*nxrB*) nucleotide sequences of both new *Nitrospira* were queried against the nr nucleotide database using BLAST, revealing low similarities to the described species. The genome of *Ca*. N. *neuquenensis* E2OT contains a *nxrA* with an identity of 86.8%–91.8% to the *nxrA* genes of the enrichment Kam‐Ns4a, and the *nxrA* of *Nitrospira* sp. Vd2 is 91.4% identical to *Ca*. *N. bockiana*. Since neither of the new *Nitrospira* genomes included *nxrB* genes, the respective sequences were obtained by employing the *Nitrospira*‐specific primer pairs 169f/638r and 19f/1237r (Pester et al. 2014). The *nxrB* of *Ca*. N. *neuquenensis* E2OT showed highest similarity to *Nitrospira* Kam‐Ns4a (94.1%) and the Uzon hot spring clones X‐8 and X‐10 (KC884906 and KC884907, respectively; 93.8% ID) (Pester et al. 2014). The *nxrB* of *Nitrospira* sp. Vd2 is closely related to the divergent *nxrB* copies of *Ca. N. bockiana* (95.1%–95.6%).

### 3.6 Genome Analysis of New *Nitrospira* Enrichments

**FIGURE 5:**
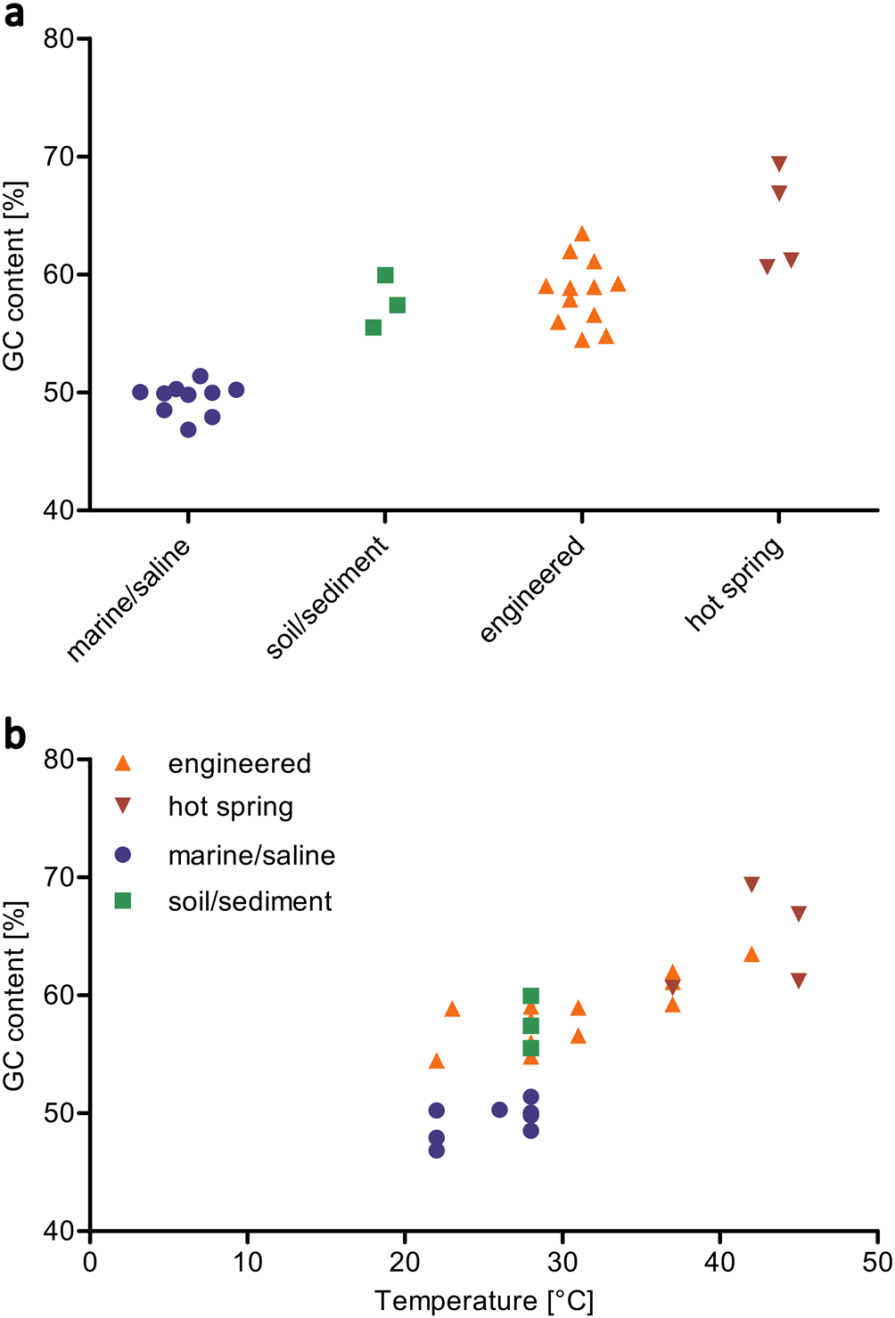
Diagrams of GC values of cultivated *Nitrospira* representatives with habitat‐specific clustering (a) or in relation to the growth temperature (b).

Metagenome sequencing of the nitrite‐oxidizing enrichments containing *Ca*. N. *neuquenensis* E2OT and *Nitrospira* sp. Vd2 resulted in high‐quality draft genomes that were assembled into 39 and 42 contigs with sizes of 3.6 and 3.9 Mbp, respectively (Table S4). The number of predicted coding sequences (CDS) was 3646 (E2OT) and 4090 (Vd2). The comparison of these MAGs with other thermophilic *Nitrospira* (*N. tepida, Ca. N. bockiana, N. calida* and *Nitrospira* sp. Kam‐Ns4a) revealed 868 (E2OT) and 1190 (Vd2) genome‐specific CDS, while 36.7%–48.5% of the CDS in the genomes were shared among these *Nitrospira* (Table S5). Based on coverage analysis of the obtained MAGs, *Ca*. N. *neuquenensis* E2OT enriched from mud sample E2 was the most abundant bacterium in the investigated culture. When grown at 42°C, it occurred together with a *Blastocatellia*‐like bacterium (phylum *Acidobacteriota*) and different *Alphaproteobacteria* (Table S6). Several MAGs from *Alphaproteobacteria*, *Gammaproteobacteria* and *Chloroflexota* were obtained from the metagenome of Vd2, but no other nitrite oxidizing bacterium could be identified. Notably, the GC content of the genome of *Nitrospira* sp. Vd2 was 60.6%, and 69.4% for the genome of *Ca*. N. *neuquenensis* E2OT, the highest value obtained so far for *Nitrospira* (Table S7). A high GC content of more than 60% seems to be typical for cultivated *Nitrospira* derived from hot springs and warm engineered systems (Figure 5).

The genomes of *Ca*. N. *neuquenensis* E2OT and *Nitrospira* sp. Vd2 contain key features conserved in the genus *Nitrospira*, like the nitrite‐oxidizing machinery, the respiratory chain for energy conservation, and the reverse tricarboxylic acid (rTCA) cycle for CO_2_ fixation (Table S8; Supporting Information: Results and Discussion). When comparing selected metabolic features of both new *Nitrospira* with the genomes of other moderately thermophilic *Nitrospira* cultures of lineage V (*Ca*. *N. bockiana* 42C) and VIII (Kam‐Ns4a), as well as the type strains of lineage VI (N. *calida* Ns10) and VII (*N. tepida* DNF; Figure S15), we observed distinct differences in their potential for energy conservation. Whereas the genomes of *Ca*. *N. bockiana* and *N. tepida* encode three different terminal oxidases and two ATPases, the two Argentinian cultures have a reduced modularity in their respiratory chain gene repertoire, putatively indicating reduced metabolic flexibility. *Ca*. N. *neuquenensis* E2OT and *Nitrospira* sp. Vd2 possess an oxygen‐reducing terminal cytochrome *bd*‐like oxidase (CytAA′, OR‐N) conserved in all *Nitrospira*, but no high‐affinity cytochrome *cbb*_*3*_ terminal oxidase. In contrast to *Ca*. N. *neuquenensis* E2OT and Kam‐Ns4a, *Nitrospira* sp. Vd2 and *Ca. N. bockiana* additionally encode a canonical cytochrome *bd*‐type quinol oxidase (CydAB). Another electron acceptor might be nitrate as alternative to oxygen, since the NXR of *Nitrospira* is known to reduce nitrate to nitrite under anoxic conditions (Koch et al. 2015). Notably, *Nitrospira* sp. Vd2 encodes a cytochrome *c* ammonia‐forming nitrite reductase (NrfAH). In addition, the nitrite reductase of N. *calida* is only distantly related to NrfA of the enrichment Vd2 (30% identity), and the genome lacks NrfH, similar to *N. tepida*. All analysed *Nitrospira* representatives have the genetic repertoire to use cyanate as an alternative N source, and they also possess urease to produce ammonium through urea hydrolysis except *N. tepida* (Figure S15, Table S8). The capability for assimilatory nitrite reduction to ammonia seems to be mediated by the membrane‐associated, periplasmic octaheme cytochrome *c* (OCC) nitrite reductase in all analysed *Nitrospira*. The cytoplasmic ferredoxin‐dependent nitrite reductase (NirA) that is present in *N. tepida* was not found in the genomes of *Nitrospira* sp. Vd2 and the enrichment E2OT.

For protection against reactive oxygen species (ROS), the *N. tepida* genome contains two types of superoxide dismutases (SODs). Contrastingly, the other *Nitrospira* in lineages V and VI encode only one SOD, and both members of lineage VIII lack this enzyme. One common feature of all investigated *Nitrospira* genomes is the presence of a cytochrome *c* peroxidase. Furthermore, a putative rubrerythrin is present in all moderately thermophilic *Nitrospira* and might support ROS defence (Table S8).

**FIGURE 6:**
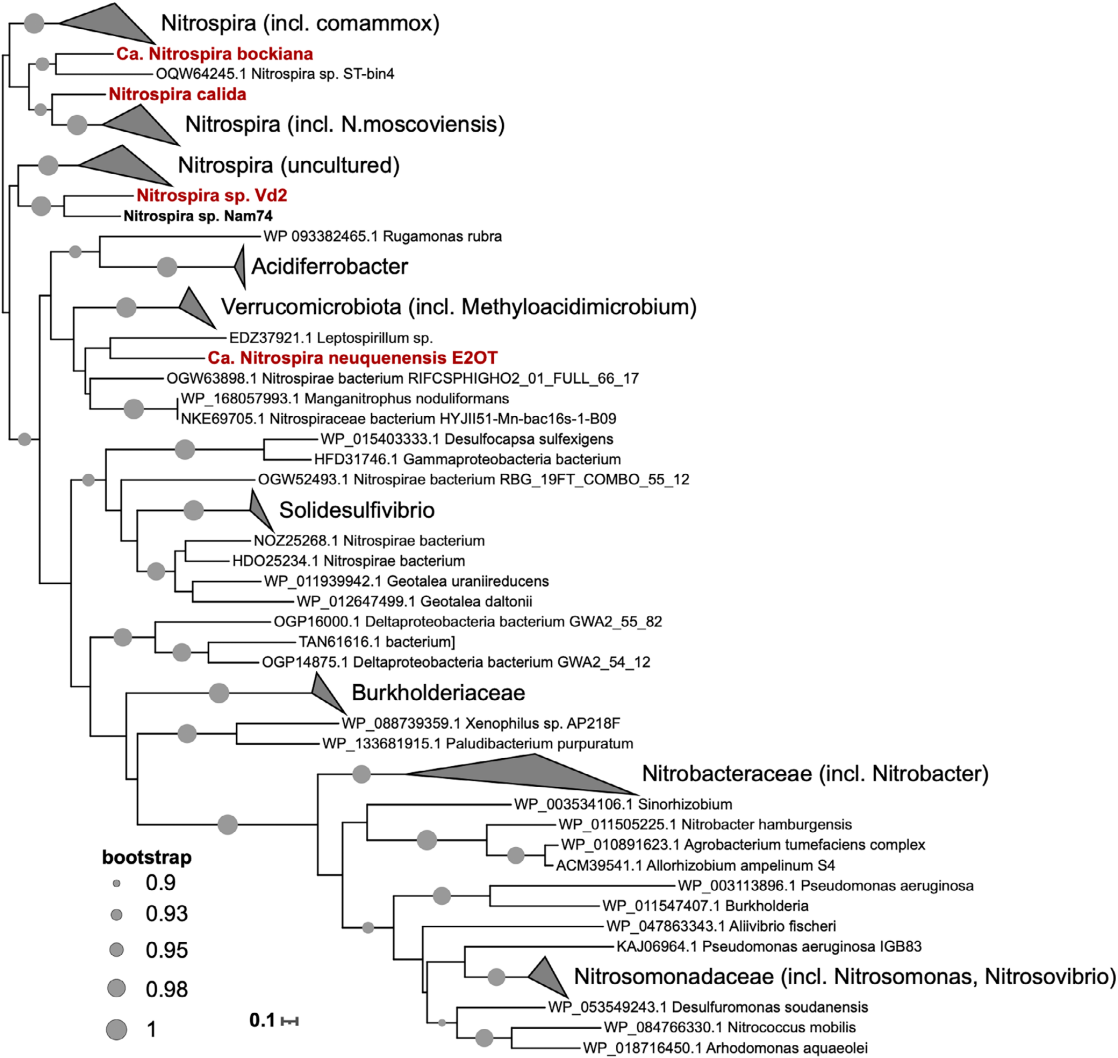
Phylogeny of AHL synthase focusing on *Nitrospira*. Maximum likelihood tree indicating the affiliation of the AHL synthase identified in the different *Nitrospira*. Statistical branching support values are indicated on the nodes. *Ca*. N. *neuquenensis* E2OT and *Nitrospira* sp. Vd2 as well as other moderately thermophilic *Nitrospira* species are shown in red. *Nitrospira* cultures not belonging to lineage II are bold. The scale bar represents the expected changes per amino acid. The root was placed at the mid‐point of the tree. For more details see Figure S16.

The genomes of *Ca*. N. *neuquenensis* E2OT and *Nitrospira* sp. Vd2 encode putative acyl homoserine lactone (AHL) synthase and a LuxR family transcriptional regulator (quorum‐sensing system regulator CviR) associated with quorum sensing (QS). Phylogenetic analysis of the AHL synthase shows that the protein sequence of Vd2 clusters with lineage II *Nitrospira*, forming a monophyletic clade (Figures 6 and S16) with 41.1% sequence identity to the corresponding protein in N. *moscoviensis* . Among lineage V, the AHL synthase of Vd2 is closely related to that of Nam74 (61.3%) but differs from the corresponding protein of *Ca*. *N. bockiana* (47.8%), which is also indicated by the clustering of these proteins into separate groups. In contrast, the AHL synthase of *Ca*. N. *neuquenensis* E2OT does not cluster with other *Nitrospira* sequences, but with a protein in *Leptospirillum* group II bacteria identified in acid mine drainage (47.9% protein sequence identity; Simmons et al. 2008), AHL synthases of *Ca. Manganitrophus noduliformans* and unclassified MAGs. This suggests a distinct evolutionary history of the QS system of *Ca*. N. *neuquenensis* E2OT compared to other *Nitrospira* AHLs.

## 4 Discussion

### 4.1 *Nitrospira*: A Common NOB in Geothermal Settings

According to current knowledge, *Nitrospira* are the most widespread NOB globally (Daims et al. 2016), inhabiting both moderate and extreme environments, including geothermal habitats (Lebedeva et al. 2011; Lebedeva et al. 2005). Based on molecular analyses, canonical and comammox *Nitrospira* are found in hot springs, including the Las Máquinas area investigated here (KP204488; Urbieta, González‐Toril, et al. 2015; Zhang et al. 2023). The aim of this study was to enrich and to genetically and physiologically characterize canonical NOB originating from the acidic geothermal area of Copahue. The identification of several *Nitrospira* as well as *Nitrolancea* species (Spieck, Sass, et al. 2020) in the same samples from Las Máquinas indicates functional redundancy of nitrite oxidation in this environment.

Several environmental drivers that serve as selection pressures to enrich novel *Nitrospira* species have already been identified and applied successfully. These include high temperatures (Lebedeva et al. 2011), extreme pH (Daebeler et al. 2020; Kim et al. 2025), low DO (Keuter et al. 2023), as well as high salinity (Keuter et al. 2011; Watson et al. 1986). In addition, the supplied substrate concentration also shapes *Nitrospira* communities (Gruber‐Dorninger et al. 2015; Maixner et al. 2006). Furthermore, the tolerance against nitrate, the end product of nitrite oxidation, is another niche‐defining factor for distinct *Nitrospira* species (Nowka et al. 2015; Sun et al. 2022). In this study, a reduced temperature of 28°C was used for the initial incubation of a sample from a geothermal spring to successfully enrich *Ca*. N. *neuquenensis* E2OT, a novel *Nitrospira* species. However, for the continued growth of this species, the temperature was raised to 37°C–42°C, as it had been from the start to enrich *Nitrospira* sp. Vd2 (42°C).

### 4.2 Cultivation Bias: Selection of *Ca.* N. *neuquenensis*

As shown by 16S rRNA gene amplicon sequencing, *Ca*. N. *neuquenensis* E2OT could not be detected in the initial 42°C enrichments. It grew neither in the initial culture of E2 (Figure S4), in which *Nitrospira* sp. Vd2 proliferated, nor in the initial NOB culture of sample A4, in which *Nitrolancea* occurred in high abundance (see below; Spieck, Sass, et al. 2020). However, *Ca*. N. *neuquenensis* E2OT could be selectively enriched at suboptimal temperatures of 28°C and 50°C, where it outcompeted *Nitrospira* sp. Vd2. Incubation at 28°C—clearly below the range for optimal growth—seems counterintuitive, since elevated temperatures were previously used to selectively enrich the new lineage VIII *Nitrospira* sp. Kam‐Ns4a, as well as moderately thermophilic *Nitrospira* from lineages V, VI and VII (Keuter et al. 2023; Lebedeva et al. 2011; Lebedeva et al. 2008). These findings revealed that incubations at suboptimal temperatures can also lead to the successful enrichment of novel *Nitrospira* species, as already shown for *Nitrospira lenta* (Nowka et al. 2015).

### 4.3 Enrichment of *Nitrospira* sp. Vd2: Competition With *Nitrolancea*

The *Ca. N. bockiana*‐like enrichment Vd2 is well adapted to the standard NOB medium without supplements and static incubation at 42°C. The relative abundance of this canonical *Nitrospira* in nitrite‐oxidizing cultures inoculated with mud from sampling site E2 drastically increased from 4% in the beginning to 27% after only one transfer, as shown by 16S rRNA gene amplicon surveys (Figure S4). However, it should be noted that these cultures were started more than 2 years (E2) or 4 years (A4) after sampling. During storage of the mud and water samples at room temperature, the samples were not amended with nitrite, indicating that *Nitrospira* sp. Vd2 is capable of surviving long periods of starvation (see below) or possesses some yet unknown alternative energy metabolism.

In contrast to sample E2, almost no *Nitrospira* were present in initial enrichments from the mud pod A4 at 42°C (Spieck, Sass, et al. 2020). Instead, previous molecular screenings revealed the dominance of *Nitrolancea* (Spieck, Sass, et al. 2020), likely due to the supplementation of ammonium to all transferred NOB cultures incubated at 37°C, supporting the growth of *Nitrolancea*, which lack the capability for assimilatory nitrite reduction. Nevertheless, long‐term cultivation at 42°C of the same sediment material in mineral NOB medium without ammonium finally led to the dominance of *Nitrospira* sp. Vd2, similar to earlier enrichment strategies (Lebedeva et al. 2011; Lebedeva et al. 2008).

### 4.4 Phylogenetic Affiliations

Phylogenomic analysis and 16S rRNA gene phylogeny revealed that *Nitrospira* sp. Vd2 clusters together with *Ca. N. bockiana* within lineage V (Figures 1 and 4). A recent genome analysis by Kop et al. (2025) extended the available genome data for additional thermophilic *Nitrospira* and proposed a new lineage VIII, which includes the enrichment culture Kam‐Ns4a (Lebedeva et al. 2011). Based on our analyses, *Ca*. *N. neuquenensis* E2OT also belongs to this new lineage, together with the hot spring enrichment Ga3a, for which only a 16S rRNA gene sequence is available.

The lineage cut‐off within the genus *Nitrospira* is defined at a 16S rRNA gene sequence identity > 94.9% to members within a lineage, and < 94% to members of other monophyletic *Nitrospira* lineages (Daims et al. 2011). Despite the 16S rRNA gene identity being slightly higher at 95% when compared to lineage VI N. *calida* , 16S rRNA gene phylogeny showed that *Ca*. N. *neuquenensis* E2OT belongs to the monophyletic and distinct *Nitrospira* lineage VIII. This was confirmed by phylogenomic analysis.

Most available *Nitrospira* genomes belong to lineages I, II and IV, whereas high‐quality genomes from lineages V to VIII are rare. Therefore, both new cultures from Argentina fill a gap in the diversity of genome‐sequenced NOB and extend the collection of (moderately) thermophilic *Nitrospira*. Since the new lineage VIII contains other *Nitrospira* enrichments and a MAG (BCRBG_21608) from hot springs (Figures 1 and 4), we suggest that its members may be well adapted to elevated temperatures, as reflected in their high genome GC contents of 63.8%–69.4%. Solely a MAG from a wastewater treatment plant (SPIREOTU_00841708) has a lower GC content of 58.7% (Table S3).

Interestingly, several *Nitrospira* sp. Vd2‐related environmental 16S rRNA gene sequences (Figure 1) originate from dry habitats such as the Atacama Desert, Chile (JF706684; Neilson et al. 2012), the Mongolian steppe (LC349263) and a semi‐arid mining site (EF612393; Mendez et al. 2008). This finding suggests that *Nitrospira* lineage V includes representatives adapted to extreme conditions, particularly dry soils, rather than high temperatures. This criterion also applies to the lineage V‐affiliated enrichment culture Nam74 obtained from Namibian soil. Notably, this enrichment was originally classified as lineage I *Nitrospira* (Nowka et al. 2015). However, after dormancy for 4 years, the dominant *Nitrospira* representative shifted to the moderately thermophilic lineage V, although biomass was enriched at 28°C (Kop et al. 2025). These observations support the oligotrophic lifestyle of *Nitrospira* lineage V members. The detection of similar *nxrB* sequences (93.4% DNA identity) in nutrient‐poor savanna soils (KX160265; Rughöft et al. 2016) may suggest that lineage V *Nitrospira* are adapted to nutrient (especially N) scarcity and water limitations. This hypothesis aligns well with the storage (and starvation) time of sample E2 and A4, which were 2–4 years before cultivation at 42°C, resulting in the enrichment of *Nitrospira* sp. Vd2.

### 4.5 Morphology and Physiology of *Ca.* N. *neuquenensis*

*Ca*. N. *neuquenensis* E2OT differs in cell morphology from the other species of this genus. In general, and as indicated by the genus name, *Nitrospira* are characterized by a spiral morphology, although most cells are only loosely twisted. In contrast, cells of *Ca*. N. *neuquenensis* E2OT possess a more vibrioid cell shape, occasionally forming short chains after cell division and might extend to variable, long rods (Figure S9b). Despite these different morphologies, only a single *Nitrospira* 16S rRNA gene sequence belonging to *Ca*. N. *neuquenensis* E2OT was detected in the investigated cultures.

Based on our observations, temperature is a niche‐defining factor between the two novel *Nitrospira* species. *Ca*. N. *neuquenensis* E2OT seems to be better adapted to lower temperatures in the initial enrichment phase, whereas higher temperatures at the beginning resulted in the enrichment of Vd2, which revealed nitrite‐oxidizing activity between 22°C and 45°C similar to *Ca*. *N. bockiana* (28°C–44°C; Lebedeva et al. 2008). However, E2OT could also be enriched using a high temperature of 50°C, indicating that this *Nitrospira* species is more competitive at the lower and upper end of the temperature spectrum compared to *Nitrospira* sp. Vd2. Using a low nitrite concentration of 0.5 mM, slow growth was observed for *Ca*. N. *neuquenensis* E2OT even at 17°C (not shown). The survival of *Ca*. N. *neuquenensis* E2OT at 50°C might be supported by its high GC content (see below) and such high temperature tolerance has been reported so far only for N. *calida* (Lebedeva et al. 2011). However, a potential influence of accompanying microorganisms on the metabolism and growth of these *Nitrospira* spp. needs to be considered, since these nitrite‐oxidizing cultures are not pure. The different starting materials of sample E2 and A4 and enrichment strategies likely resulted in different side communities. Whereas the metagenome of *Ca*. N. *neuquenensis* , as well as a 50°C culture inoculated with follow‐up cultures of sample E2, contained sequences belonging to *Acidobacteriota* and different *Alphaproteobacteria* in addition to *Nitrospira*, the bacterial communities of the enrichments derived from sample A4 were more complex. The presence of additional bacteria belonging to the phyla *Actinomycetota* and *Chloroflexota* and the class *Deltaproteobacteria* may support nitrite oxidation at high temperatures, probably through the exchange of vitamins or cofactors (Bayer et al. 2021; Kim et al. 2025; Xu et al. 2022). While the two *Nitrospira* were the only known NOB within the cultures, nitrite‐oxidizing activity by a yet unknown NOB within the side community cannot be ruled out.

### 4.6 High GC Content as a Potential Adaptation to Elevated Temperature

Temperature was identified as a strong driving force for adaptive evolution (Sauer and Wang 2019), and a high GC content was suggested to lead to increased thermostability in bacteria (Hu et al. 2022; Nishio et al. 2003; Shu and Huang 2022). Despite some criticism of this theory (Wang et al. 2006), it seems reasonable that growth temperature is one of the factors influencing the genomic GC content of aerobic bacteria (Musto et al. 2006), but thermal adaptation is also conferred through additional genomic features (Zeldovich et al. 2007). Following the reverse ecological principle, the optimal growth temperature of a bacterium can be predicted based on its genome sequence. For N. *moscoviensis* , 38.2°C was calculated (Sauer and Wang 2019), which correlates well with laboratory experiments (Ehrich et al. 1995).

One of the most striking features of the novel *Ca*. N. *neuquenensis* E2OT is its extraordinarily high GC content of 69.4%, which is even higher than that of *Nitrospira* Kam‐Ns4a (66.9%) belonging to the same novel lineage VIII (Table S7). Most *Nitrospira* species have a significantly lower GC content, particularly those from marine or saline environments and colder habitats (Table S7; Figure 5b; Mueller et al. 2023). Although all moderately thermophilic *Nitrospira* species except for *Ca*. N. *inopinata* (59.2%) have a GC content above 60% (Table S7), no lineage‐specific conservation of this evolutionary trait was apparent. Instead, there may be a habitat‐specific clustering of the GC content of cultivated *Nitrospira* (Figure 5a). Lineage V *Nitrospira* including the enrichment Vd2 (60.6% GC) is also characterized by high GC contents with the exception of the soil enrichment culture Nam74 (55.5% GC) which has a lower optimal growth temperature (28°C) than the other lineage V members (37°C–42°C; Table S7). In accordance, genomes of other thermophilic nitrite‐oxidizing groups also have high GC values (Spieck, Spohn, et al. 2020, 2020a) as well as thermophilic comammox MAGs (Zhang et al. 2023). Still, the relationship between GC content and habitat remains unclear for uncultivated *Nitrospira*, (Figure S17, Table S3).

### 4.7 Metabolic Properties Based on Genome Analysis

Our study confirms variability in the respiratory chain and nitrogen assimilation mechanisms among moderately thermophilic *Nitrospira*, as recently analysed in a broader context (Kop et al. 2025). Surprisingly, the nitrite‐oxidizing consortium of *Ca*. N. *neuquenensis* E2OT grew well under shaking, which is normally not used for the cultivation of *Nitrospira*, because they often lack the classical defence mechanisms against oxidative stress (Lücker et al. 2010). While both new Argentinian representatives contain peroxidases in their genomes, only Vd2 encodes an SOD. However, all moderately thermophilic *Nitrospira* encode rubrerythrin. This ferritin‐like protein is believed to have evolved in a microaerobic and thermophilic environment (Cardenas et al. 2016) and has been linked to oxidative stress tolerance in anaerobic bacteria and archaea (Sztukowska et al. 2002). Additionally, the accompanying heterotrophic bacteria may protect *Nitrospira* from ROS. As another environmental stress defence mechanism, the genomic inventory of *Nitrospira* E2OT and Vd2 includes the capability to produce spermidine. This polyamine is involved in the acid stress response of *Leptospirillum* (Vergara et al. 2020) and may help *Nitrospira* cope with the extreme conditions in their natural habitat.

### 4.8 Quorum Sensing: Typical in Moderately Thermophilic *Nitrospira*?

The genomes of both *Nitrospira* E2OT and Vd2 encode putative AHL synthases and LuxR family transcriptional regulators associated with QS. To date, AHL‐based QS systems have mainly been described in lineage II *Nitrospira* (Mellbye et al. 2017; Sakoula et al. 2018; Ushiki et al. 2018), including comammox (Sun et al. 2018; Wang et al. 2021). However, the distribution of QS was recently found to extend to other *Nitrospira* cultures within lineages V and VI, but not yet to lineage VIII (Kop et al. 2025). The AHL synthase of *Ca*. N. *neuquenensis* E2OT clusters separately from other *Nitrospira* lineages in the tree and forms a distinct branch together with unclassified *Nitrospira* and non‐nitrifiers (Figure 6). The role of QS in *Nitrospira* is still unknown, but Mellbye et al. (2016) suggested that it regulates the flux of nitrogen oxides during nitrification in *Nitrobacter*. Additionally, it has been postulated that AHL synthesis influences cell adhesion, biofilm formation and nitrifying activity (Li et al. 2015; Ma, Cheng, et al. 2023; Sun et al. 2018). Furthermore, AHL may enhance the competitiveness of nitrifying bacteria under adverse environmental conditions, such as low temperatures (Cui et al. 2026; Qiu et al. 2025; Zeng and Hu 2023), but the role of AHL in adapting to increasing temperatures has not been investigated. Although the AHLs of the new *Nitrospira* cultures have not yet been analysed biochemically, the identification of AHL synthase genes in these genomes expands the known distribution of QS systems in the genus *Nitrospira*.

### 4.9 Description of “Candidatus *Nitrospira neuquenensis*” sp. nov.

Nitrospira neuquenensis (neu.quen.en'sis. N.L. fem. adj. neuquenensis), pertaining to the Copahue region in Neuquén, North Patagonia, Argentina.

The enrichment culture *Nitrospira* E2OT originated from a hot spring in the geothermal field Las Máquinas, Neuquén‐Argentina. It is a moderately thermophilic, aerobic, chemolithoautotrophic bacterium that stoichiometrically converts nitrite to nitrate and uses carbon dioxide as a carbon source. Young cultures form short vibrioid rods, measuring 0.35–0.55 μm in diameter and 1–2 μm in length, which can extend to long, flexible rods with lengths of up to 8.4 μm under high substrate concentrations or during long‐term feeding. Cells are mobile, Gram‐negative and do not form biofilm or microcolonies. The optimum growth temperature is 42°C, with a growth range between 22°C and 50°C. Based on the genome sequence, the GC content is 69.4 mol%.

## 5 Conclusions

In this study, we used temperature stress at the lower and upper growth limits (28°C and 50°C) in combination with manual cell separation to selectively enrich a distinct novel *Nitrospira* species, and we provide genomic data for understudied lineages within the family *Nitrospiraceae*. While the most ubiquitous and diverse *Nitrospira* lineages I, II and IV are well represented by both cultivated species and environmental MAGs, cultures and genomes of lineages V to VIII are still scarce, highlighting the importance of both cultivation‐based and molecular approaches. Cultivated representatives of these lineages often occupy very specific niches, as found in geothermal systems (both engineered and natural) and warm wastewater. The selective enrichment of two novel *Nitrospira* in this study expands the known diversity of NOB in thermal habitats. All *Nitrospira* cultures with a high GC content (above 60%) grow well at elevated temperatures (≥ 37°C), indicating that this feature may contribute to their thermostability.

## Author Contributions

E.D., A.G. and W.S. performed sampling. E.S. undertook cultivation. E.S. and M.M. conducted research. H.K., L.F.M.K., K.S., S.L. and P.P.G. analysed data. E.S., S.K., H.K., S.L., L.F.M.K. and A.G. wrote the paper. H.K., S.K., M.M., L.F.M.K., K.S. prepared figures. H.K., S.K., E.S. and M.M. revised the manuscript.

## Funding

This work was supported by Deutsche Forschungsgemeinschaft, SP 667/7‐1, SP 667/7‐2, SP 667/11‐1, SP 667/11‐2; Austrian Science Fund (FWF; https://doi.org/10.55776/COE7); Nederlandse Organisatie voor Wetenschappelijk Onderzoek, VI.Veni.192.086; SIAM (Soehngen Institute of Anaerobic Microbiology), 024.002.002.

## Ethics Statement

All prevailing local, national and international regulations and conventions and normal scientific ethical practices have been respected.

## Conflicts of Interest

The authors declare no conflicts of interest.

## Supporting information


**Figure S1:** Geographical map of the sampling site Las Máquinas in the Copahue geothermal area, Neuquén province, Argentina. The map was created using ArcGIS Pro (Esri Inc. ArcGIS Pro Version 3.1.7.).
**Figure S2:** Overview of the geothermal location in Argentina. (a) photograph of thermal waters in Las Máquinas and details of the origin of samples E2 (b) and A4 (c).
**Figure S3:** Mud and water samples of Las Máquinas and enrichment scheme of *Nitrospira* sp. Vd2 and *Ca. N. neuquenensis* E2OT. Various incubation temperatures resulted in selective growth of different *Nitrospira* (only one of them was detectable at the time of investigation). Red = E2OT, yellow = Vd2, green = N. *japonica* . Culture vessels represent a series of follow‐up cultures at the same incubation temperature. OT = optical tweezer, 0.45 μm filter size.
**Figure S4:** Composition of bacterial phyla and *Nitrospira* sp. Vd2 based on 16S rRNA amplicon sequences. Nitrite‐oxidizing enrichments (0.3 mM nitrite) from sample E2 were inoculated in 2011 and grown at 42°C. DNA of culture E2.1 (03.02.2011) was extracted in April 2015, E2.2 (24.03.2015) in August 2015 and E2.3 (23.03.2016) in May 2016.
**Figure S5:** Growth of the initial nitrite‐oxidizing enrichment derived from mud sample E2. Temperature optimum was analysed 2010 with 0.8 mM nitrite, the test was inoculated with cells grown at 28°C. Optimal nitrite consumption at different temperatures was evaluated between day 0 and 12.
**Figure S6:** High degree of enrichment of *Ca*. N. neuquenensis E2OT grown at 37°C with 5 mM nitrite as substrate under shaking. (a) Genus‐specific probe Ntspa662, Cy3, (b) EUB I‐III, FITC, (c) Dapi, (d) overlay.
**Figure S7:** Composition of the bacterial community of two 50°C cultures of *Ca. N. neuquenensis* E2OT based on 16S rRNA amplicon sequencing. The nitrite‐oxidizing consortium A4_50 (derived from water sample A4) was incubated with 3 mM nitrite. The nitrite oxidizing culture E2_50 (derived from mud sample E2) was incubated with 0.5 mM nitrite and resulted from the dilution step 10^−3^.
**Figure S8:** Fluorescence in situ hybridization of *Ca*. N. neuquenensis E2OT in a 50°C nitrite‐oxidizing culture (0.5 mM nitrite) derived from sample A4. (a) specific probe Ntspa662, Cy3, (b) EUB I‐III, FITC, (c) Dapi, (d) overlay.
**Figure S9:** Variability in cell length of *Ca. N. neuquenensis* E2OT in dependence on the consumed substrate. (a) Short curved rod, grown with 0.5 mM of nitrite with shaking and stained after 6 weeks, (b) “S‐form” and long flexible rod. The culture was grown at 42°C with 5 mM nitrite and additionally supplied with a high amount of substrate when consumed. Staining occurred after 6 weeks. Incubation was done without shaking and biomass was used for genome sequencing. (c) Flagella‐wearing vibrioid‐like cell, grown in the presence of 3 mM substrate and incubated on a shaker for 3 weeks.
**Figure S10:** Cell morphology and ultrastructure of *Nitrospira* sp. Vd2. (a) Negatively stained short twisted rod, (b) Ultrathin section of a microcolony.
**Figure S11:** Fluorescence in situ hybridization of *Nitrospira* sp. Vd2. (a) Genus‐specific probe Ntspa662, cy3, (b) EUB I‐III, FITC, (c) Dapi, (d) overlay.
**Figure S12:** Growth of *Ca. N. neuquenensis* E2OT derived from mud sample E2. (a) Temperature optimum was analysed 2023 with 1.4 mM nitrite, the test was inoculated with cells grown at 37°C. The values represent the average of three biological replicates with the exception of 45°C, where growth only occurred in two replicates. Optimal nitrite consumption at different temperatures was evaluated between day 3 and 10. (b) Nitrite oxidation at 42°C. Determination of nitrite and nitrate of separate flasks started on day 3, start values were measured in the same batch.
**Figure S14:** Average nucleotide and average amino acid identities between the recovered *Nitrospira* genomes and the genomes of cultivated *Nitrospira* representatives.
**Figure S15:** Genomic comparison of *Ca. N. neuquenensis* E2OT and *Nitrospira* sp. Vd2 with other moderately thermophilic *Nitrospira* species in lineage V‐VIII focusing on selected metabolic key features. QS, quorum sensing; ROS, reactive oxygen species; TO, terminal oxidase; OCC, octaheme cytochrome *c*; CynS, cyanase; NirA, ferredoxin‐dependent nitrite reductase; NirK, NO forming nitrite reductase; NrfAH, ammonia forming nitrite reductase; SOD, superoxide dismutase; cyt., cytochrome; RBR, rubrerythrin; AHL, acyl‐homoserine lactone. More information can be found in Table S8. * nrfA and nrfH don't form a gene cluster, ** Due to a frameshift in the gene sequence, it is not clear whether a functional protein can be expressed.
**Figure S16:** Phylogeny of AHL synthase focusing on *Nitrospira*. Maximum likelihood tree indicating the affiliation of the AHL synthase identified in the different *Nitrospira* strains. Statistical branching support values are indicated on the nodes. *Ca*. N. *neuquenensis* E2OT and *Nitrospira* sp. Vd2 as well as other moderately thermophilic *Nitrospira* species are shown in red. The root was placed at the mid‐point of the tree. The scale bar represents the expected changes per amino acid.
**Figure S17:** GC values (%) of uncultivated *Nitrospira* MAGs sorted by their habitats. Data are based on Kop et al. (2025). Habitat categories are according to Table S3.

**Figure S13:** Phylogenomic classification of *Ca*. N. *neuquenensis* E2OT and *Nitrospira* sp. Vd2 based on the concatenated alignment of 71 conserved bacterial marker proteins. The genomes of *Ca*. N. *neuquenensis* E2OT and *Nitrospira* sp. Vd2 are labelled in bold. Names of *Nitrospirales* genomes included in GlobDB R226 and the prefix “Candidatus” was omitted. Black circles indicate bootstrap support of 100%.

**Table S1:** 16S rRNA gene sequences obtained by amplicon sequencing and their taxonomical assignments.

**Table S2:** Accession number of 16S rRNA gene sequences and genome sequences used for phylogenetic tree calculation.

**Table S3:** GC content of *Nitrospira* MAGs.
**Table S4:** General characteristics of the draft genomes of *Ca. N. neuquenensis* E2OT and *Nitrospira* sp. Vd2.

**Table S5:** Pangenome analysis comparing *Ca. Nitrospira neuquenensis* E2OT, *Nitrospira* sp. KamNS4, *Nitrospira calida* Ns10, *Nitrospira* sp. Vd2, *Ca. Nitrospira bockiana* 47C and *Nitrospira tepida* using the pangenome tool of genoscope and following parameters: 50% amino acid identity and 80% alignment coverage.

**Table S6:** General characteristics of MAGs binned from the two metagenomes.

**Table S7:** GC content of cultivated *Nitrospira* species.

**Table S8:** Predicted coding sequences (CDS) and comparison of selected metabolic features of *Ca*. N. *neuquenensis* E2OT and *Nitrospira* sp. Vd2 with other moderately thermophilic *Nitrospira* species.

## Data Availability

Genome data of *Ca*. N. *neuquenensis* E2OT and *Nitrospira* sp. Vd2 are available at the European Nucleotide Archive (PRJEB98483), 16S rRNA and *nxrAB* sequences at the NCBI (PX369882, PX369883, PX417423‐PX417426) and Zenodo (https://doi.org/10.5281/zenodo.17235877).
