# Supplementary material for: Cultivation‐Based Detection of a Novel High‐GC Nitrospira Derived From the Argentinian Copahue Volcano Area
Source: Environ Microbiol. 2026 Apr 3;28(4):e70290. doi: 10.1111/1462-2920.70290 (PMC13049253; doi:10.1111/1462-2920.70290)
Supplement: Supplementary file 1 — Figure S1: Geographical map of the sampling site Las Máquinas in the Copahue geothermal area, Neuquén province, Argentina. The map was created using ArcGIS Pro (Esri Inc. ArcGIS Pro Version 3.1.7.). Figure S2: Overview of the geothermal location in Argentina. (a) photograph of thermal waters in Las Máquinas and details of the origin of samples E2 (b) and A4 (c). Figure S3: Mud and water samples of Las Máquinas and enrichment scheme of Nitrospira sp. Vd2 and Ca. N. neuquenensis E2OT. Various incubation temperatures resulted in selective growth of different Nitrospira (only one of them was detectable at the time of investigation). Red = E2OT, yellow = Vd2, green = N. japonica . Culture vessels represent a series of follow‐up cultures at the same incubation temperature. OT = optical tweezer, 0.45 μm filter size. Figure S4: Composition of bacterial phyla and Nitrospira sp. Vd2 based on 16S rRNA amplicon sequences. Nitrite‐oxidizing enrichments (0.3 mM nitrite) from sample E2 were inoculated in 2011 and grown at 42°C. DNA of culture E2.1 (03.02.2011) was extracted in April 2015, E2.2 (24.03.2015) in August 2015 and E2.3 (23.03.2016) in May 2016. Figure S5: Growth of the initial nitrite‐oxidizing enrichment derived from mud sample E2. Temperature optimum was analysed 2010 with 0.8 mM nitrite, the test was inoculated with cells grown at 28°C. Optimal nitrite consumption at different temperatures was evaluated between day 0 and 12. Figure S6: High degree of enrichment of Ca. N. neuquenensis E2OT grown at 37°C with 5 mM nitrite as substrate under shaking. (a) Genus‐specific probe Ntspa662, Cy3, (b) EUB I‐III, FITC, (c) Dapi, (d) overlay. Figure S7: Composition of the bacterial community of two 50°C cultures of Ca. N. neuquenensis E2OT based on 16S rRNA amplicon sequencing. The nitrite‐oxidizing consortium A4_50 (derived from water sample A4) was incubated with 3 mM nitrite. The nitrite oxidizing culture E2_50 (derived from mud sample E2) was incubated with 0.5 mM nitrite and [file EMI-28-e70290-s003.docx]

**Supplementary Material**

**Cultivation-based detection of a novel high-GC *Nitrospira* derived from the Argentinian Copahue volcano area**

Eva Spieck^1*^, Hanna Koch^2,3,4^, Linnea F.M. Kop^3^, Sabine Keuter^1,5^, Marcel Malinowski^1^, Katharina Sass^6^, Wolfgang Sand^7,8^, Edgardo Donati^9^, Pablo Perez Garcia^1^, Sebastian Lücker^3^, Alejandra Giaveno^10^

^1^ Department of Microbiology and Biotechnology, Institute of Plant Sciences and Microbiology, University of Hamburg, Ohnhorststr. 18, 22609 Hamburg, Germany

^2^ Bioresources Unit, Center for Health & Bioresources, AIT Austrian Institute of Technology GmbH, Konrad-Lorenz-Straße 24, 3430 Tulln an der Donau, Austria

^3^ Department of Microbiology, Radboud Institute for Biological and Environmental Sciences, Radboud University, Heyendaalseweg 135, 6525 AJ, Nijmegen, the Netherlands

^4^ Division of Microbial Ecology, Centre for Microbiology and Environmental Systems Science, University of Vienna, Djerassiplatz 1, 1030 Vienna, Austria

^5^ Institute of Carbon Cycles, Helmholtz-Zentrum Hereon GmbH, Max-Planck-Straße 1, 21502 Geesthacht, Germany

^6^ Institute for General Microbiology, Christian-Albrechts-Universität zu Kiel, Am Botanischen Garten 1-9, 24118 Kiel, Germany

^7^ Aquatic Biotechnology, Faculty of Chemistry, University Duisburg-Essen, Universitätsstr. 5, 45141 Essen, Germany

^8^ Institute for Oceanology of the Chinese Academy of Sciences in Qingdao, Shandong, PR China

^9^ CINDEFI (CCTLaPlata-CONICET, UNLP), Facultad de Ciencias Exactas, Universidad Nacional de La Plata, Calle 47 y 115, 1900 La Plata, Argentina

^10^ PROBIEN (CONICET-UNCo), Departamento de Química, Facultad de Ingeniería, Universidad Nacional del Comahue. Neuquén, Argentina

**Supplementary methods**

*Amplicon sequencing*

For the enrichments E2.1 and E2.2, 10 ng of genomic DNA were used to generate 16S rRNA gene amplicons using the primers 341F and 785R (*Herlemann et al.*, 2011) followed by 454 pyrosequencing (300 bp, 3000 reads per sample). For culture E2.3, the primers 515F and 806R (*Caporaso et al.*, 2011) were used, with subsequent Illumina MiSeq sequencing (2 × 300 bp, 20,000 reads per sample) by MR DNA (MR DNA, Shallowater, TX, USA). Operational taxonomic units (OTUs) were obtained from the preprocessed sequences using the Qiime (v1.9.1) de novo OTU picking workflow and summarized and visualized using the summarize_taxa_through_plots.py script (*Caporaso et al.*, 2010). Illumina sequences were processed, classified, and summarized by the analysis pipeline of MR DNA (*Dowd et al.*, 2008). In short, sequences were joined and depleted of barcodes, followed by the removal of sequences <150 bp or with ambiguous base calls. Sequences were denoised, OTUs generated, and chimeras removed. OTUs were defined by clustering at 3% divergence (97% similarity). Final OTUs were taxonomically classified using BLASTn against a curated database derived from NCBI and RDPII ([www.ncbi.nlm.nih.gov](http://www.ncbi.nlm.nih.gov/), [http://rdp.cme.msu.edu](http://rdp.cme.msu.edu/)).

16S rRNA amplicon sequencing of the E2OT enrichment from sample A4 grown at 50 °C was performed at Eurofins (Ebersberg, Germany) by Illumina sequencing (NovaSeq, 5M read pairs) with the bacterial V3/V4 16S rRNA primers 347F and 800R (*Kisand et al.*, 2002; *Turner et al.*, 1999). First, all reads with ambiguous bases (“N”) and chimeric reads were identified and removed using the de-novo algorithm of UCHIME (*Edgar et al.*, 2011), as implemented in the VSEARCH package (*Rognes et al.*, 2016). Subsequently, the remaining reads were further processed and partitioned into OTUs (Operational Taxonomic Units) using Minimum Entropy Decomposition (MED) (*Eren et al.*, 2015, 2013). For taxonomic assignment of the OTUs, DC-MEGABLAST searches against a sequence database were performed. Taxonomic classification required a minimum sequence identity of 70% across at least 80% of the representative sequence. Further processing of the OTUs and their taxonomic assignments was carried out using the QIIME software package (version 1.9.1, <http://qiime.org/>). Sequences with very low abundance (less than approximately 0.02% of the sample’s total reads) were removed.

16S rRNA amplicons of the *Nitrospira* enrichment E2OT from the sample E2 grown at 50 °C (A10) were ordered at Novogene (Munich, Germany) with the bacterial V3/V4 16S rRNA primers 341F and 806R. Size-selected PCR products were pooled in equimolar amounts, end-repaired, A-tailed, ligated to Illumina adapters, and sequenced as paired-end reads on an Illumina platform (PE250, 50 K). Raw reads were merged, quality-filtered, and chimera-checked to generate effective tags, which were clustered into OTUs at 97% sequence identity. Taxonomic assignment was conducted using the SILVA 138.2 reference database to determine microbial community composition and relative abundance.

*Metagenomic sequencing and assembly*

Metagenome sequences of the enrichment culture E2OT were edited as follows: Quality trimming and filtering of raw reads were performed using BBDuk v38.8 (*Bushnell*, 2014), and quality checking was conducted with FastQC v0.11.9 (Babraham Bioinformatics) and MultiQC v1.12 (*Ewels et al.*, 2016). Assembly was performed with Spades v3.13.1 (*Prjibelski et al.*, 2020) in metagenomic mode. For binning, reads were mapped back to the contigs with BWA-MEM v0.7.17 (*Li*, 2013), and MAGs were generated with MetaBat v2.12.1 (*Kang et al.*, 2019).

Processing of metagenomic sequences of the enrichment Vd2 was done by the described steps. After incorporation of indexed adaptors and amplification, the libraries were purified using AMPure XP beads (Beckman Coulter, Indianapolis, USA). Size distribution within the resulting libraries was checked using the Agilent 2100 Bioanalyzer (Agilent Technologies, Santa Clara, USA), and library DNA concentrations were measured using the Qubit dsDNA HS Assay Kit (ThermoFisher Scientific Inc., Waltham, USA) on a Qubit (v2.0). The libraries were diluted to a final concentration of 4 nM, denatured, and paired-end sequenced (2 × 300 bp) on a MiSeq system using MiSeq reagent kit v3 (Illumina). Bbduk bbtool v.37.76 (https://jgi.doe.gov/data-and-tools/bbtools) was used to remove sequencing adaptors and to trim the raw reads with the following settings: k=23, mink=11, hdist=1, ktrim=r, tbo, qtrim=rl, maq=20, maxns=0, minlen=150, tossjunk=t and trimq = 18. The trimmed Illumina reads were assembled into contigs using Spades v3.14.0 (*Nurk et al.*, 2017) with the metagenome option and default parameters. The trimmed reads were mapped onto the obtained contigs using Burrows–Wheeler aligner (BWA) v.0.7.17-r1188 (*Li*, 2013) with the mem algorithm and default settings to obtain coverage information. For automatic binning, MetaBat2 (*Kang et al.*, 2019) was employed with the following settings: minimum contig length=1000, minimum contig depth=1, and minimum bin size=0.5 Mbp.

*Genome and ANI analyses*

The quality of both *Nitrospira* genomes was assessed using checkM2 v1.0.1 (*Chklovski et al.*, 2023). The GC values of uncultivated *Nitrospirales* MAGs (n=455; dataset obtained from *Kop et al.*, 2025; Table S3) were determined using seqkit stats (*Shen et al.*, 2024) and visualized in R (R Core Team, 2022) using the functions ‘geom_violin’ and ‘geom_force’ of the packages ggplot (v3.5.1; *Wickham*, 2016) and ggforce (v0.4.2;)(*Kim et al.*, 2021; *Pedersen*, 2025). The average nucleotide identity (ANI) between the recovered *Nitrospira* genomes and the genomes of cultivated *Nitrospira* strains was calculated using anvi’o integrated 'PyANI' by Pritchard et al. (2016). For the calculation of the average amino acid identity (AAI) the tool EzAAI – v1.2.3 was used (*Kim et al.*, 2021).

Gene calling and automatic annotation was performed using the annotation platform MicroScope (*Vallenet et al.*, 2020). The webserver-based CRISPRCasFinder (https://crisprcas.i2bc.paris-saclay.fr/) (*Couvin et al.*, 2018) with the default settings was used to predict CRISPR-Cas systems. For identification of homologs in other *Nitrospira* genomes, the tool gene phyloprofile within the MicroScope platform was used with homology constraints ≥ 35% identity, minLrap ≥ 0.8 and maxLrap ≥ 0.8. The pangenome analysis tool embedded in the GenoScope annotation platform was used to calculate the shared and unique proteins using the MICFAM parameters of 50% amino acid identity and 80% alignment coverage.

For estimating the genome completeness and contamination checkM and checkM2 was employed. While checkM1 is a well-established tool to predict genome completeness and contamination based on lineage-specific marker gene sets, its newer version (checkM2) builds models based on machine learning without considering taxonomic information and showed improved performance for certain lineages (*Chklovski et al.*, 2023) including our two *Nitrospira* genomes (Table S4).

*Phylogenic analyses*

An alignment of all 539 16S rRNA gene sequences taxonomically classified as *Nitrospira* within the Silva SSU Ref NR database (r138.1) was downloaded (arb-silva.de/browser). In addition, the 16S rRNA genes of *Nitrospira* sp. Vd2 and *Ca.* N. neuquenensis E2OT were surveyed against the NCBI nucleotide collection nr/nt using BLASTn. In total, 369 sequences were selected to calculate the 16S rRNA phylogenetic tree (Table S2) with separation into lineages based on published definitions (*Daims et al.*, 2001; *Kop et al.*, 2025; *Lebedeva et al.*, 2011). Selected 16S rRNA *Nitrospira* sequences obtained by the survey and others, including the outgroup, were added to the alignment by using the ACT tool of silva arb-silva.de/aligner/. The alignment was trimmed using trimAL v1.5.rev1 (*Capella-Gutiérrez et al.*, 2009) using the parameters -gt 0.95 -cons 60. Based on the trimmed alignment with 1567 distinct patterns, a maximum-likelihood phylogenetic tree was calculated using IQ-TREE 3.01 (*Wong,* 2025) with 1000 ultrafast bootstrap replications (*Hoang et al.*, 2018) and TNe+I+R6 as best-fitting substitution model identified via the integrated modelfinder (*Kalyaanamoorthy et al.*, 2017)

In addition, a phylogenetic tree was calculated using anvi’o dev (*Eren et al*., 2021) which included all *Nitrospirales* genomes from globDB R226 https://globdb.org/ (*Speth et al.*, 2025) nine recently published *Nitrospira* genomes (*Kop et al.*, 2025), and the genomes of Vd2 and E2OT (n=677). For calculation of the phylogenetic tree, the protein sequences of 71 conserved bacterial marker genes were extracted from all genomes, the sequences were aligned using muscle (*Edgar*, 2004) and the resulting alignments were concatenated using anvi-get-sequences-for-hmm-hits --hmm-sources Bacteria_71 --return-best-hit --unique-genes --concatenate-genes --get-aa-sequences. Subsequently, a phylogenetic tree was calculated based on the concatenated alignment containing 23,612 amino acid positions using anvi-gen-phylogenomic-tree and FastTree v2.2.0 (*Price et al.*, 2010) with Shimodaira-Hasegawa test (SH)-like support with 1000 replications. Five UBA2166 MAGs were used to root the tree.

AHL synthase sequences were extracted from the *Nitrospira* MAGs and downloaded from the publicly available non-redundant database NCBI. Protein sequences were aligned using the multiple sequence alignment program MAFFT L-INS-i v7 (*Katoh*, 2002). Subsequently, maximum-likelihood trees were calculated using anvi’o (*Eren et al.*, 2021) and displayed in iTOL v7.2.1 (*Letunic and Bork*, 2007).

**Supplementary results and discussion**

*Genome analysis of new* Nitrospira *enrichments*

The genomes sizes of E2OT and Vd2 range between 3.6 and 3.9 Mbp, similar to non-thermophilic cultivated *Nitrospira* spp. including strains from the lineages V-VIII (3.3-4.9 Mbp (*Bayer et al.*, 2021; *Kop et al.*, 2025).

*Ca.* N. neuquenensis E2OT encodes a NxrA, several putative NxrCs, and a nitrite/nitrate transporter. In the genome of *Nitrospira* sp. Vd2, no *nxrA* gene could be detected, but a *Nitrospira* *nxrA* was identified in the assembly. In addition, the *nxrB* sequences of both species could be amplified by PCR. The electrons derived from nitrite oxidation either transferred to the terminal oxidase for energy conversion or to reducing equivalent reduction for CO2 fixation via reverse electron transport. The respiratory chain complexes are conserved in *Nitrospira*, but a modularity of these complexes was observed via comparative genomics (*Kop et al.*, 2025). In our analysis, complexes I-III were well conserved in the thermophilic species, but multiple versions of complex IV and V were identified. Complex IV is discussed in the main text. The two novel representatives each contain only one ATPase of the F_o_F_1_-type, whereas *Nitrospira* sp. Kam-Ns4a, the second member of the new lineage VIII, and *Ca*. N. bockiana also encode a V-type ATPase similar to other *Nitrospira* (*Kop et al.*, 2025). A conserved feature in *Nitrospira* is the copper-containing NO-forming nitrite reductases (NirK), which was present in both Argentinian *Nitrospira* representatives.

*Nitrospira moscoviensis* can use alternative substrates such as hydrogen and formate for autotrophic growth. For this, the strain employs a 2a [NiFe] hydrogenase and a NADH-dependent fomate dehydrogenase (*Koch et al.*, 2015, 2014). Like other moderately thermophilic cultures except for *Ca.* N. bockiana and the enrichment Vd2, the genome of *Ca.* N. neuquenensis E2OT includes a group 3b [NiFe] hydrogenase potentially for oxidizing hydrogen as an alternative energy source, although its exact metabolic role is unknown. The genome of *Nitrospira* sp. Vd2 contains a region encoding the hydrogen maturation protease of group 3b [NiFe] hydrogenases and other hydrogenase proteins. However, most of the genes of the hydrogenase gene cluster are truncated, and the gamma subunit (HypB) is missing. Although the genome analysis suggests that the hydrogenase of *Nitrospira* sp. Vd2 might be not functional, its potential activity remains to be determined in future physiological experiments. Whereas *Ca.* N. bockiana encodes a *N. marina*-like formate dehydrogenase (*Kop et al.*, 2025), none of the other genomes analyzed here encode a formate-degrading enzyme system.

Both new *Nitrospira* representatives possess CRISPR loci and *cas* gene cassettes as a potential defense mechanism against phages. While Vd2 contains one *cas* gene cluster, E2OT possesses two. However, it remains to be determined whether their CRISPR-Cas systems are functional, as all *cas* cassettes may lack key genes. In contrast, *Ca.* N. bockiana does not possess any Cas system (Table S8). Similar to many described *Nitrospira* species, the genomes of the Argentinian species contained a chlorite dismutase, a spermidine synthase, and the corresponding transporter. In addition, both new *Nitrospira* can use a trehalose/maltose transporter to import the osmoprotectant trehalose, which only Vd2 seems to be able to synthesize for defense against salt stress, as proposed for other moderately thermophilic *Nitrospira* (*Kop et al.*, 2025). Furthermore, both analyzed *Nitrospira* have the capacity for motility and chemotaxis, and flagella have been found in electron micrographs of *Ca.* N. neuquenensis E2OT (Fig. S9c).

**Supplementary figures**


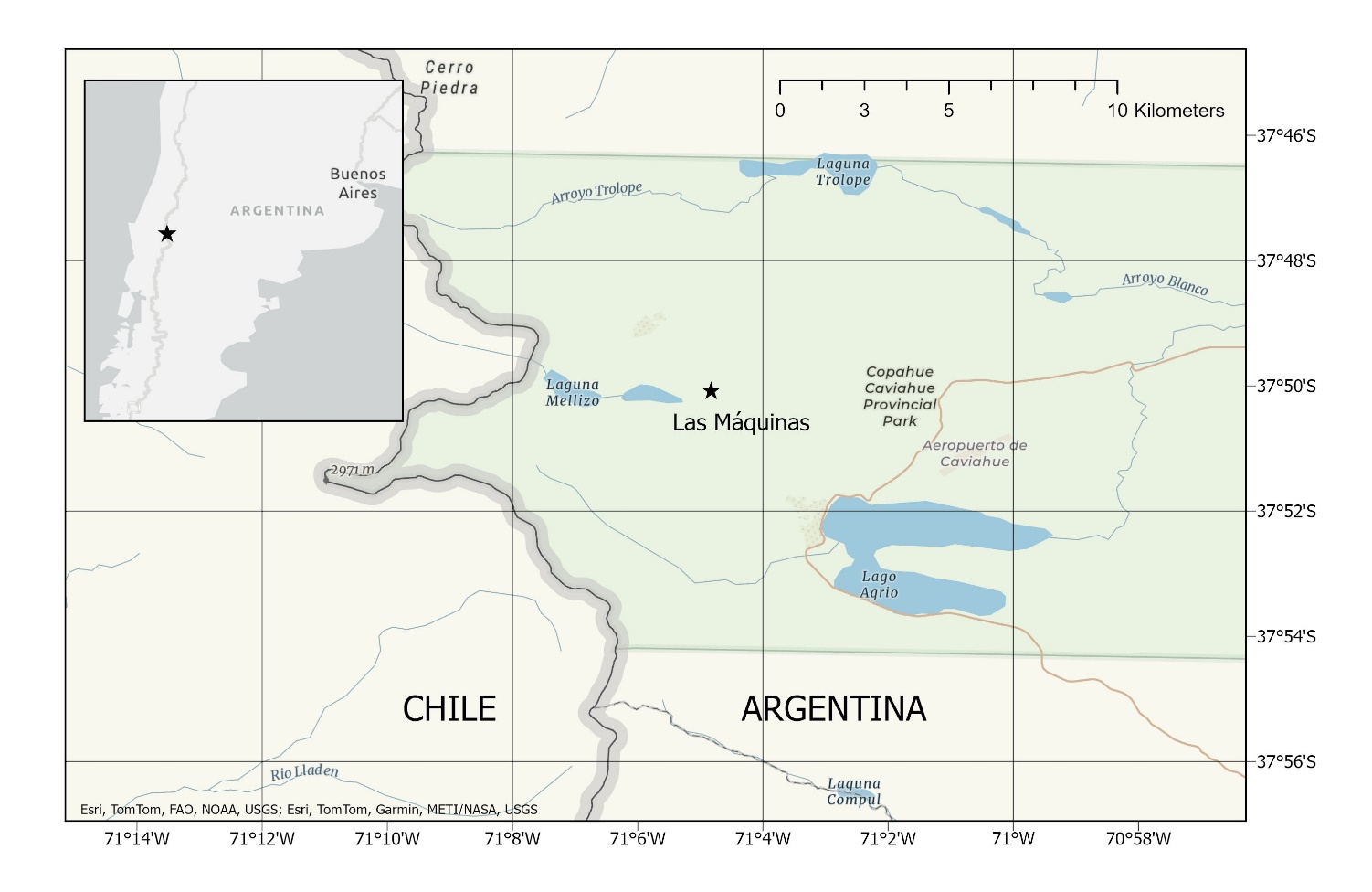
**Figure S1:** Geographical map of the sampling site Las Máquinas in the Copahue geothermal area, Neuquén province, Argentina. The map was created using ArcGIS Pro (Esri Inc. ArcGIS Pro Version 3.1.7.)


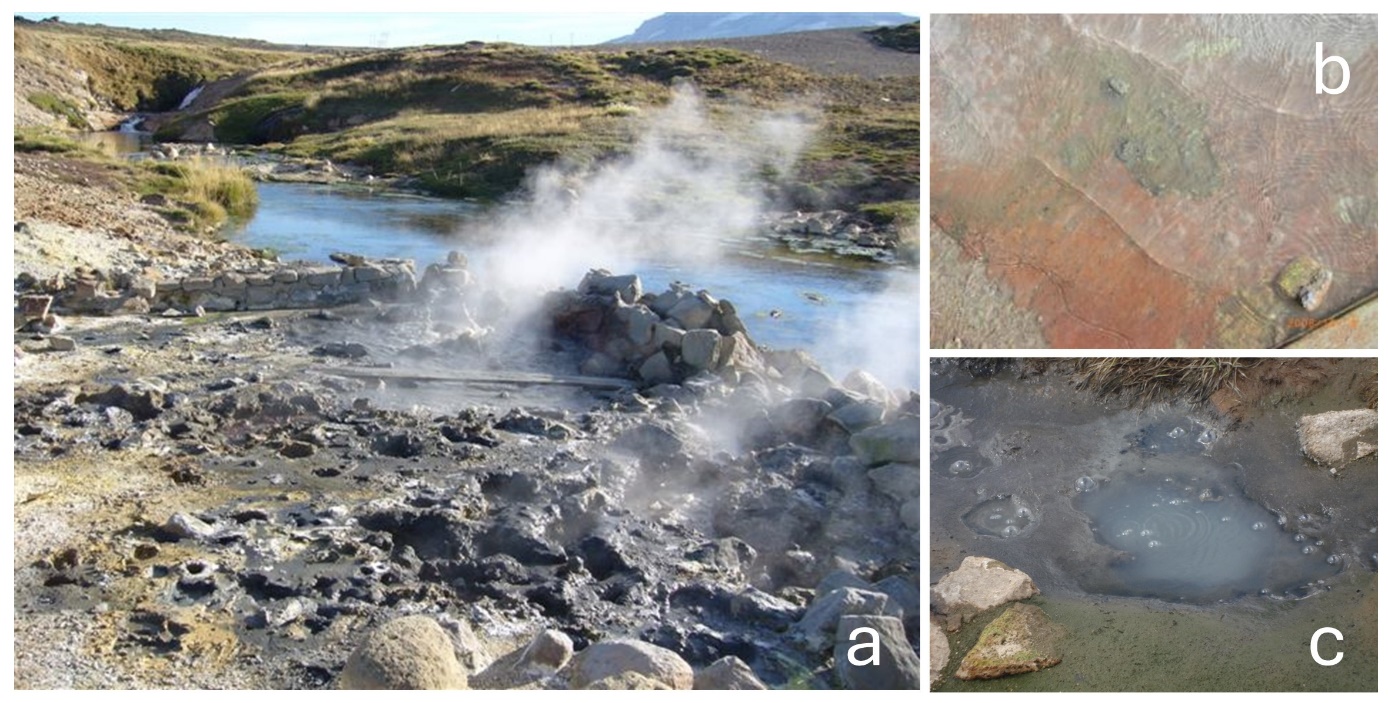


**Figure S2:** Overview of the geothermal location in Argentina. a) photograph of thermal waters in Las Máquinas and details of the origin of samples E2 (b) and A4 (c).


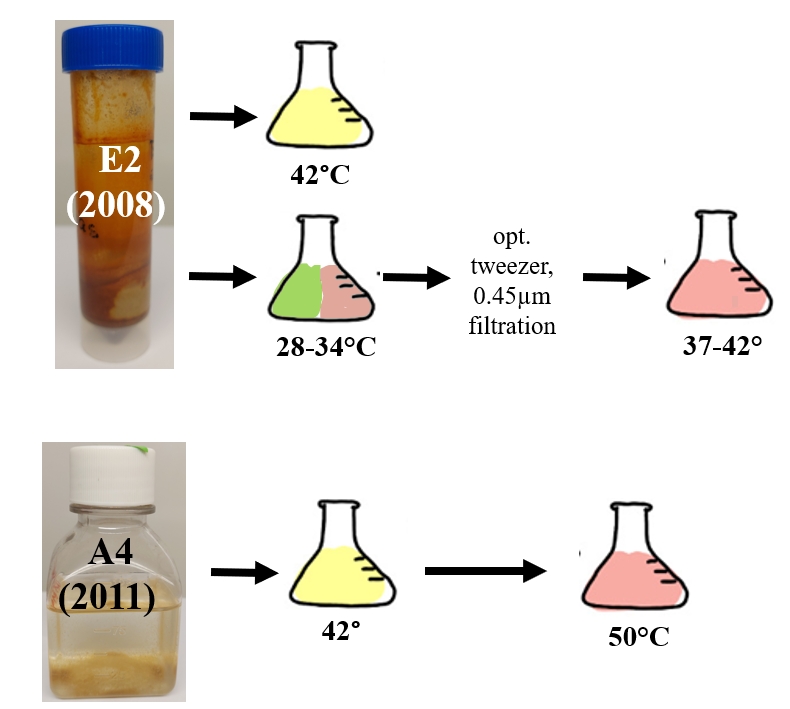


**Figure S3:** Mud and water samples of Las Máquinas and enrichment scheme of *Nitrospira* sp. Vd2 and *Ca.* N. neuquenensis E2OT. Various incubation temperatures resulted in selective growth of different *Nitrospira* (only one of them was detectable at the time of investigation). Red=E2OT, yellow=Vd2, green=*N. japonica*. Culture vessels represent a series of follow-up cultures at the same incubation temperature. OT= optical tweezer, 0.45 µm filter size.

**
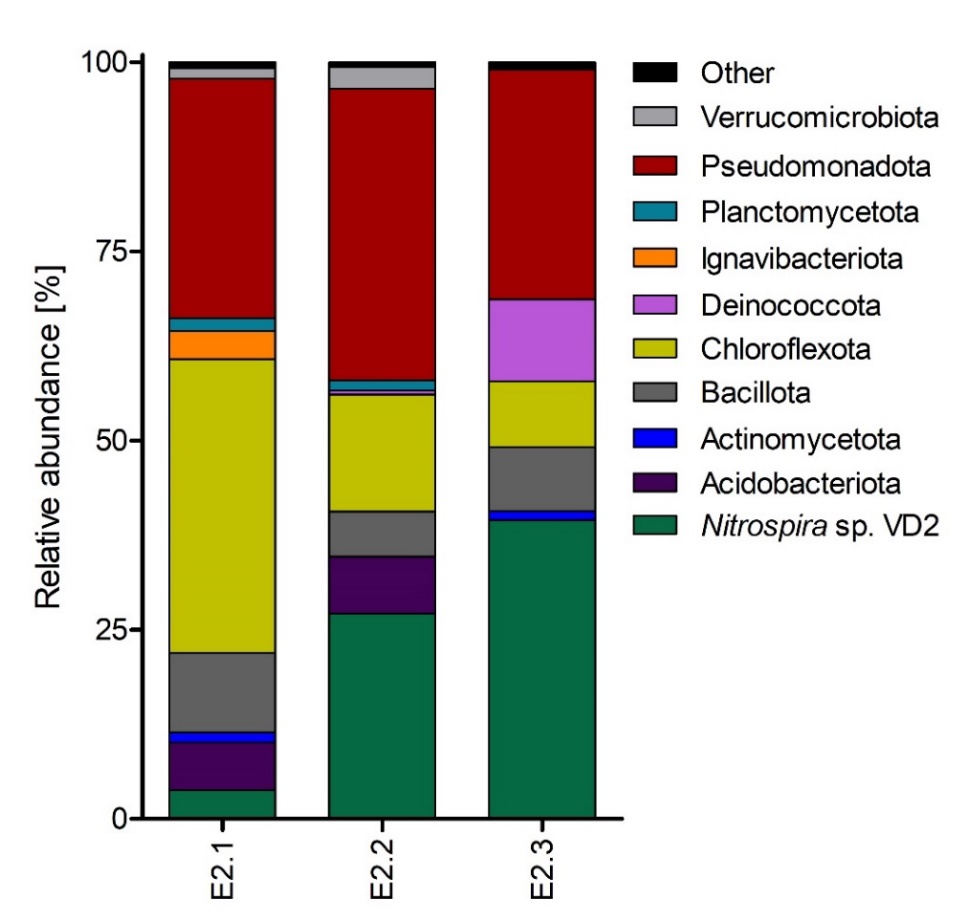
**

**Figure S4:** Composition of bacterial phyla and *Nitrospira* sp. Vd2 based on 16S rRNA amplicon sequences. Nitrite-oxidizing enrichments (0.3 mM nitrite) from sample E2 were inoculated in 2011 and grown at 42°C. DNA of culture E2.1 (03.02.2011) was extracted in April 2015, E2.2 (24.03.2015) in August 2015 and E2.3 (23.03.2016) in May 2016.

**
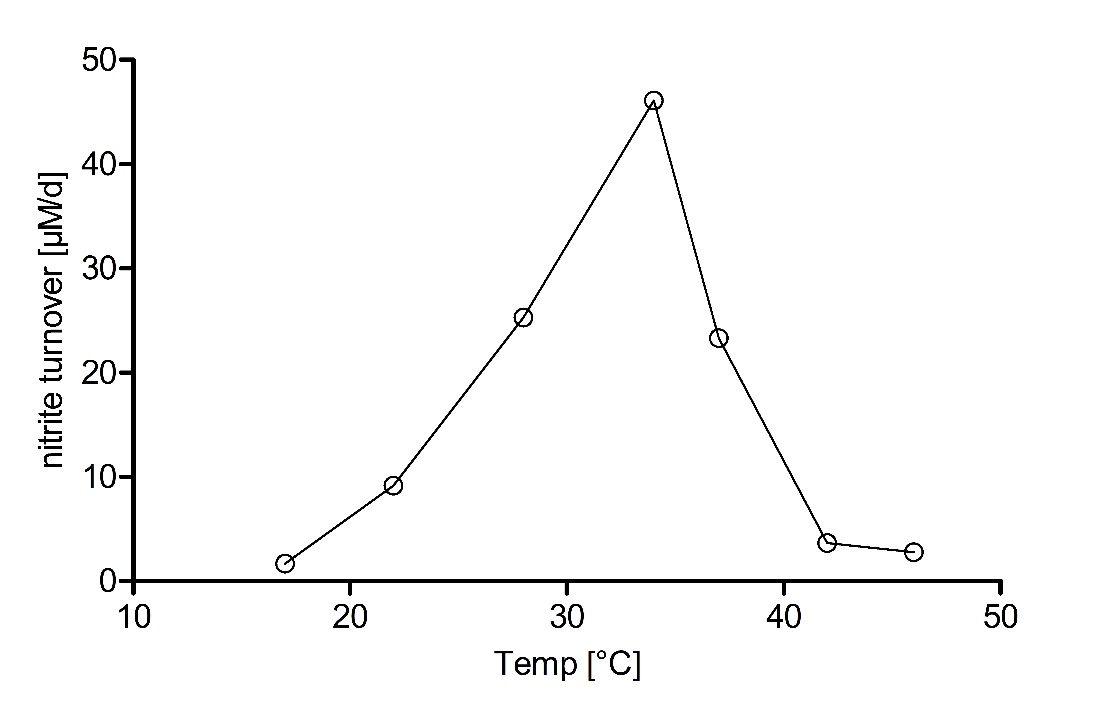
**

**Figure S5:** Growth of the initial nitrite-oxidizing enrichment derived from mud sample E2. Temperature optimum was analyzed 2010 with 0.8 mM nitrite, the test was inoculated with cells grown at 28 °C. Optimal nitrite consumption at different temperatures was evaluated between day 0 and 12.

**
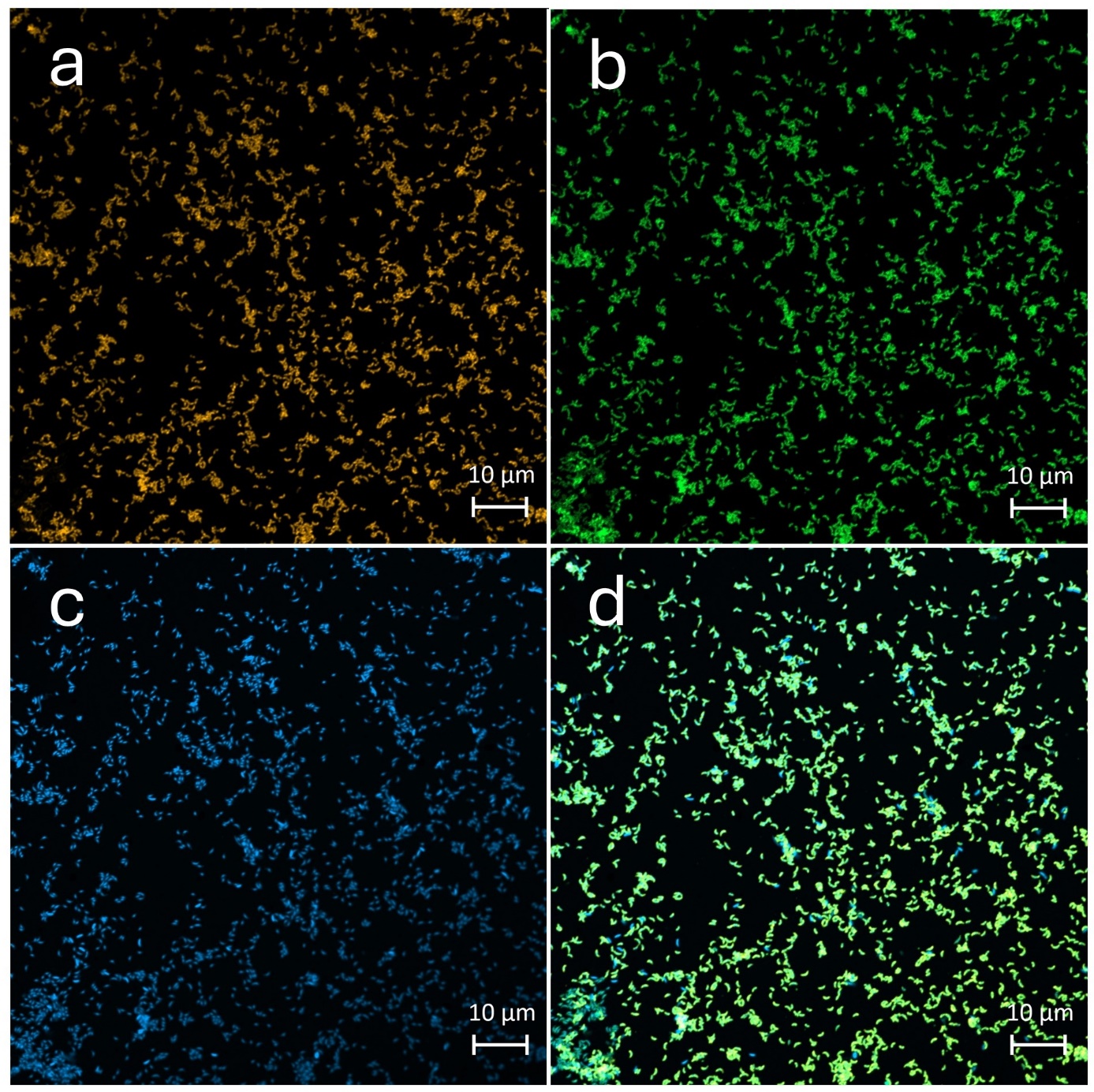
**

**Figure S6:** High degree of enrichment of *Ca*. N. neuquenensis E2OT grown at 37°C with 5 mM nitrite as substrate under shaking. a) genus-specific probe Ntspa662, cy3, b) EUB I-III, FITC, c) Dapi, d) overlay.

**
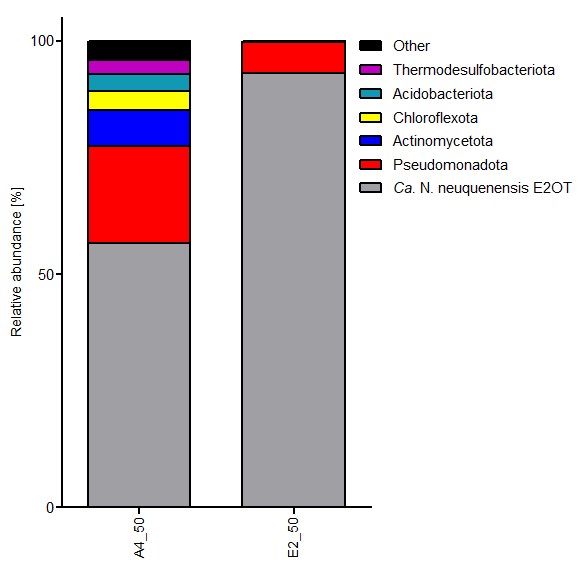
**

**Figure S7:** Composition of the bacterial community of two 50°C cultures of *Ca.* N. neuquenensis E2OT based on 16S rRNA amplicon sequencing. The nitrite-oxidizing consortium A4_50 (derived from water sample A4) was incubated with 3 mM nitrite. The nitrite oxidizing culture E2_50 (derived from mud sample E2) was incubated with 0.5 mM nitrite and resulted from the dilution step 10^-3^.


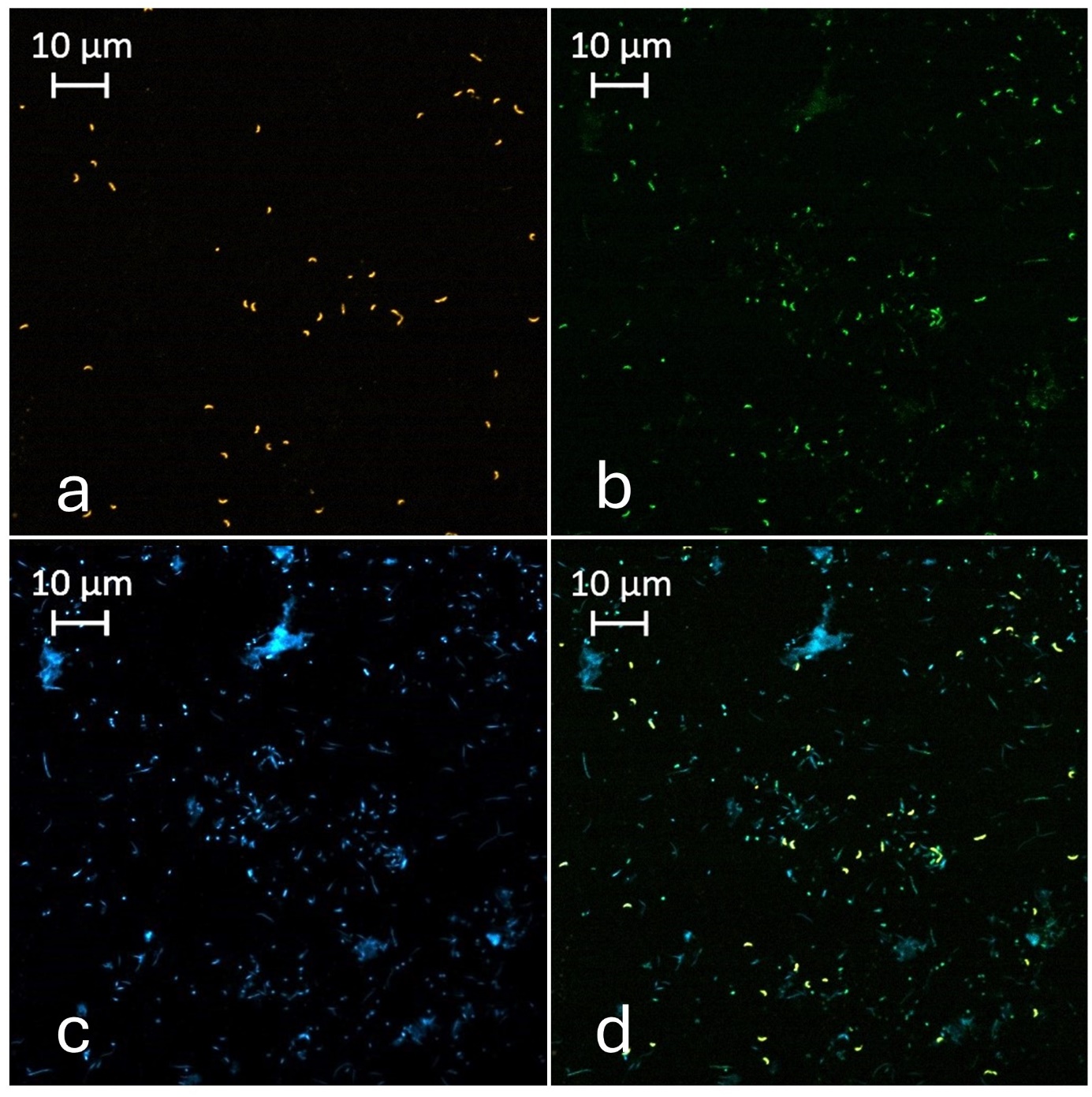


**Figure S8:** Fluorescence *in-situ* hybridization of *Ca.* N. neuquenensis E2OT in a 50°C nitrite-oxidizing culture (0.5 mM nitrite) derived from sample A4. a) specific probe Ntspa662, cy3, b) EUB I-III, FITC, c) Dapi, d) overlay.


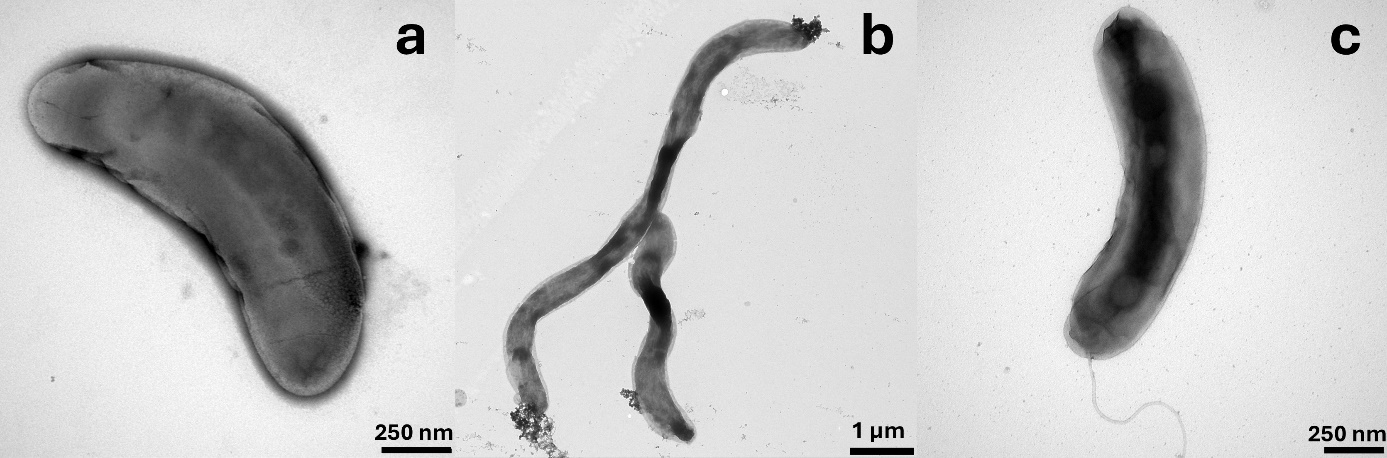


**Figure S9:** Variability in cell length of *Ca.* N. neuquenensis E2OT in dependence on the consumed substrate. a) short curved rod, grown with 0.5 mM of nitrite with shaking and stained after 6 weeks, b) “S-form” and long flexible rod. The culture was grown at 42°C with 5 mM nitrite and additionally supplied with a high amount of substrate when consumed. Staining occurred after 6 weeks. Incubation was done without shaking and biomass was used for genome sequencing. c) Flagella-wearing vibrioid-like cell, grown in the presence of 3 mM substrate and incubated on a shaker for 3 weeks.


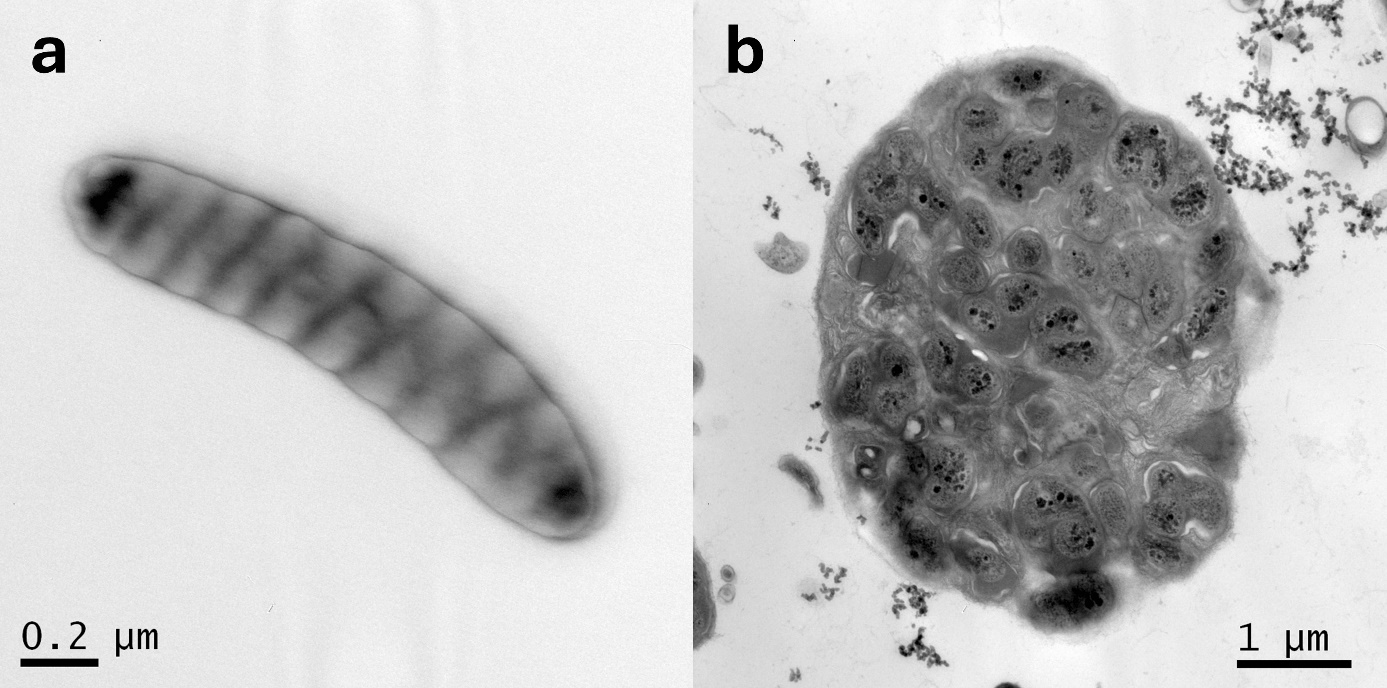


**Figure S10:** Cell morphology and ultrastructure of *Nitrospira* sp. Vd2. A) Negatively stained short twisted rod, b) Ultrathin section of a microcolony.


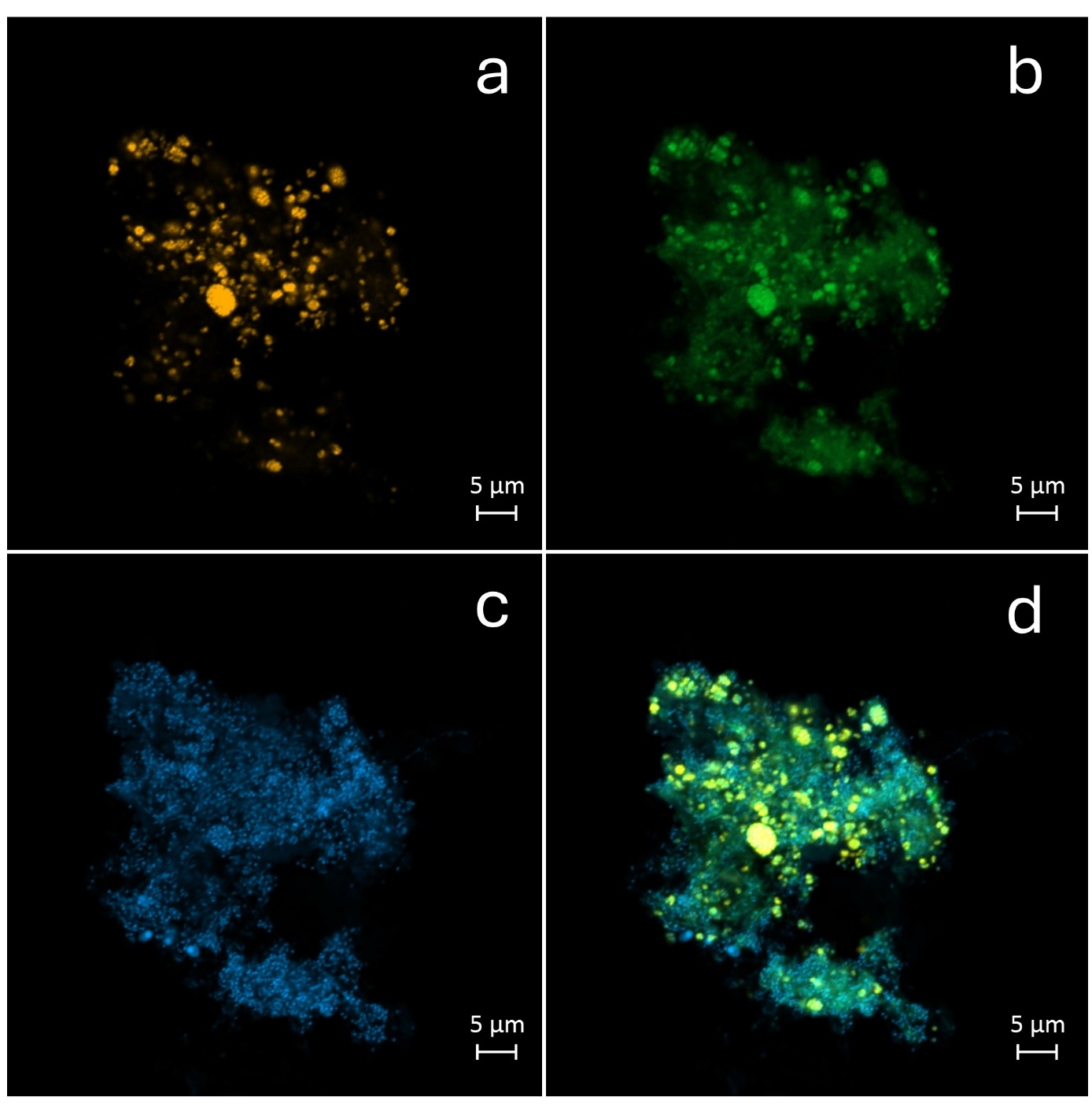


**Figure S11:** Fluorescence *in-situ* hybridization of *Nitrospira* sp. Vd2. a) genus-specific probe Ntspa662, cy3, b) EUB I-III, FITC, c) Dapi, d) overlay.

**
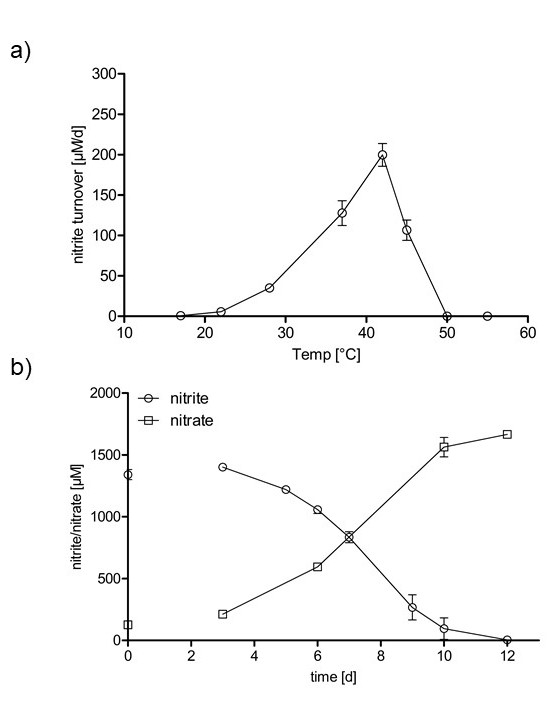
**

**Figure S12:** Growth of *Ca.* N. neuquenensis E2OT derived from mud sample E2. a) Temperature optimum was analyzed 2023 with 1.4 mM nitrite, the test was inoculated with cells grown at 37°C. The values represent the average of three biological replicates with the exception of 45°C, where growth only occurred in two replicates. Optimal nitrite consumption at different temperatures was evaluated between day 3 and 10. b) Nitrite oxidation at 42°C. Determination of nitrite and nitrate of separate flasks started on day 3, start values were measured in the same batch.

**Figure S13:** Phylogenomic classification of *Ca*. N. neuquenensis E2OT and *Nitrospira* sp. Vd2 based on the concatenated alignment of 71 conserved bacterial marker proteins. The genomes of *Ca*. N. neuquenensis E2OT and *Nitrospira* sp. Vd2 are labelled in bold. Names of *Nitrospirales* genomes included in GlobDB R226 and the prefix “Candidatus” was omitted. Black circles indicate bootstrap support of 100%. (see separate file).

**
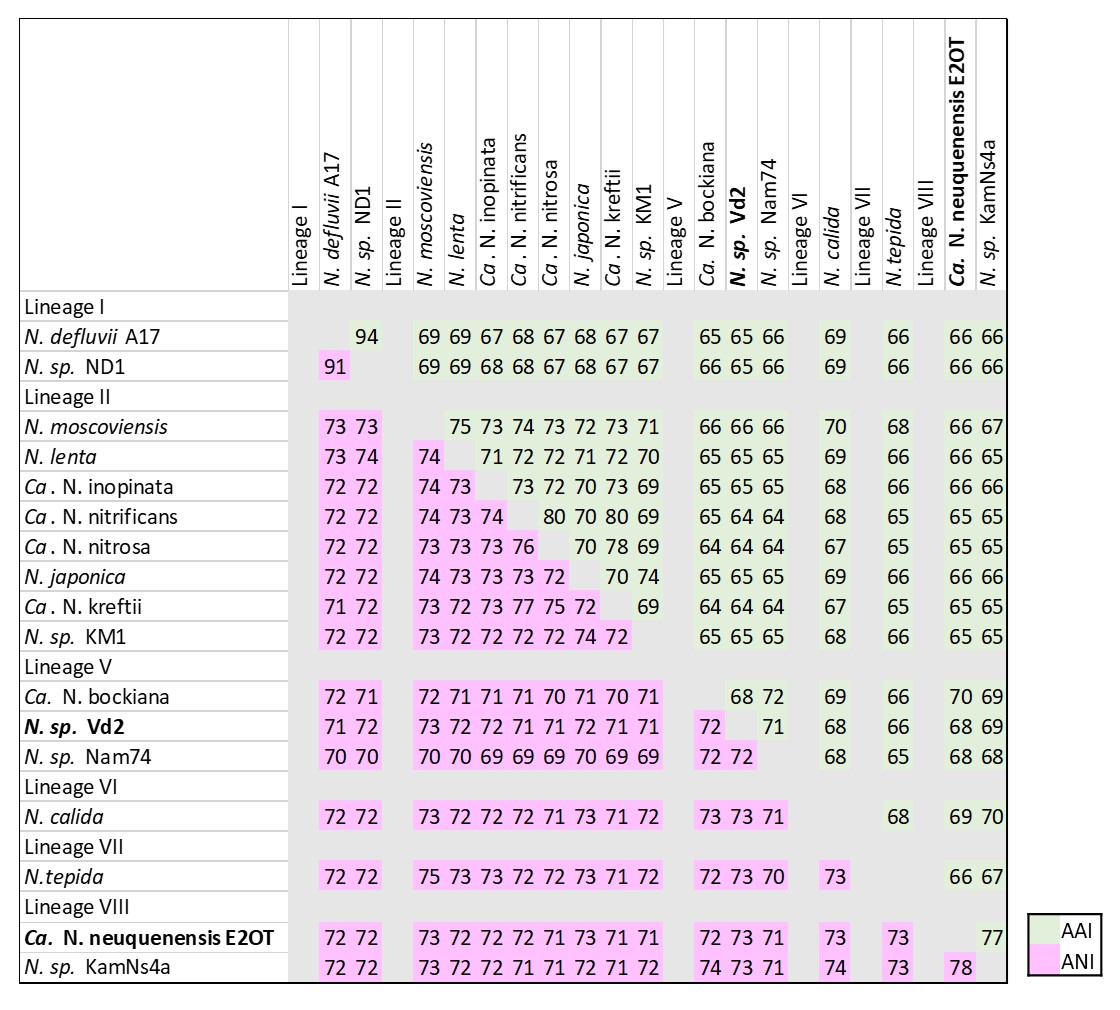
**

**Figure S14:** Average nucleotide and average amino acid identities between the recovered *Nitrospira* genomes and the genomes of cultivated *Nitrospira* representatives.

**
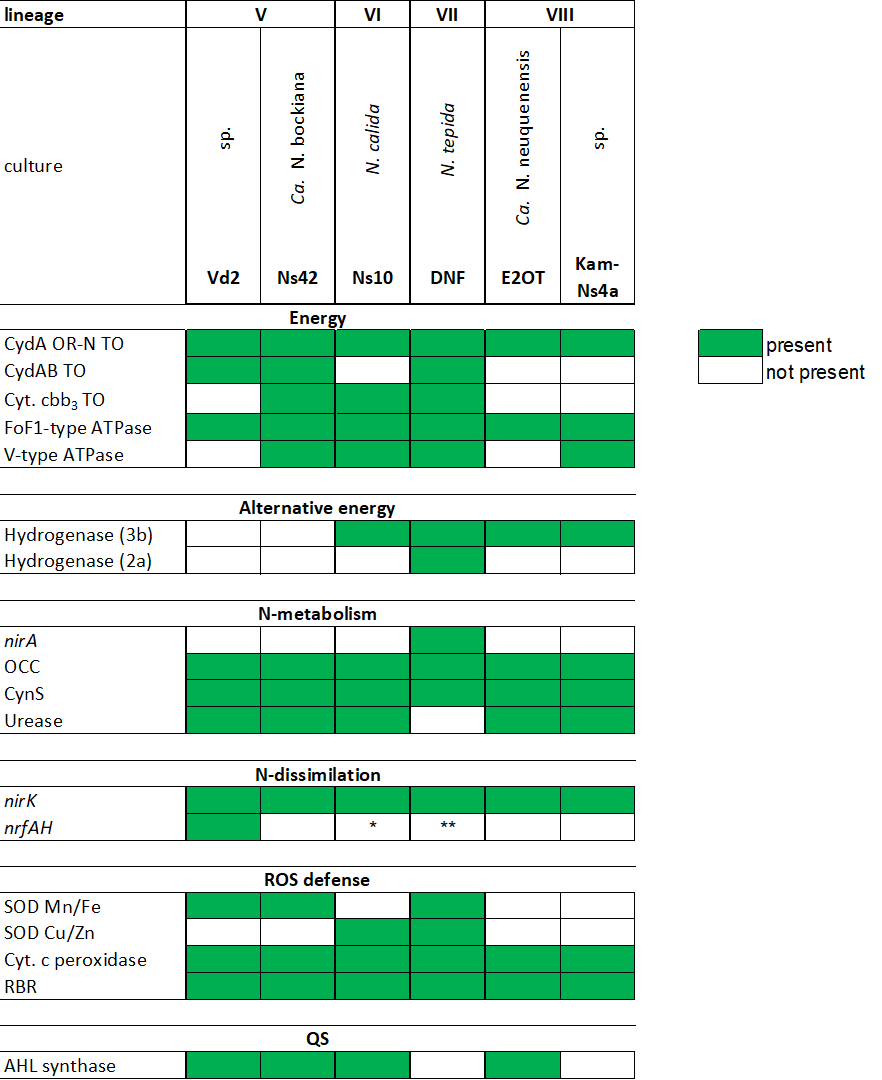
**

**Figure S15:** Genomic comparison of *Ca.* N. neuquenensis E2OT and *Nitrospira* sp. Vd2 with other moderately thermophilic *Nitrospira* species in lineage V-VIII focusing on selected metabolic key features. QS, quorum sensing; ROS, reactive oxygen species; TO, terminal oxidase; OCC, octaheme cytochrome *c*; CynS, cyanase; NirA, ferredoxin-dependent nitrite reductase; NirK, NO forming nitrite reductase; NrfAH, ammonia forming nitrite reductase; SOD, superoxide dismutase; cyt., cytochrome; RBR, rubrerythrin; AHL, acyl-homoserine lactone. More information can be found in Supplementary Table S8. * nrfA and nrfH don't form a gene cluster, ** Due to a frameshift in the gene sequence, it is not clear whether a functional protein can be expressed.

**
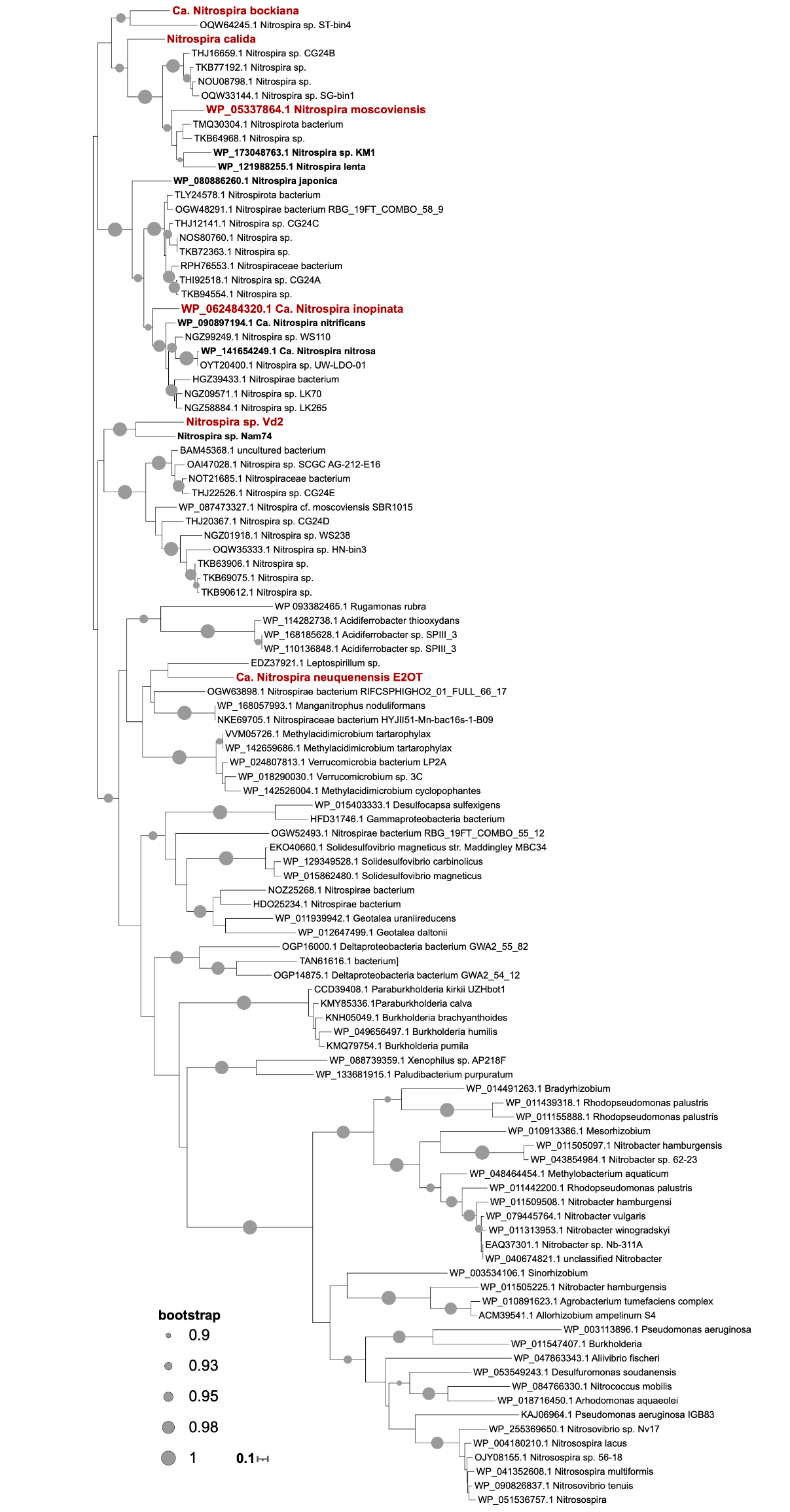
**

**Figure S16:** Phylogeny of AHL synthase focusing on *Nitrospira*. Maximum likelihood tree indicating the affiliation of the AHL synthase identified in the different *Nitrospira* strains. Statistical branching support values are indicated on the nodes. *Ca*. N. neuquenensis E2OT and *Nitrospira* sp. Vd2 as well as other moderately thermophilic *Nitrospira* species are shown in red. The root was placed at the mid-point of the tree. The scale bar represents the expected changes per amino acid.


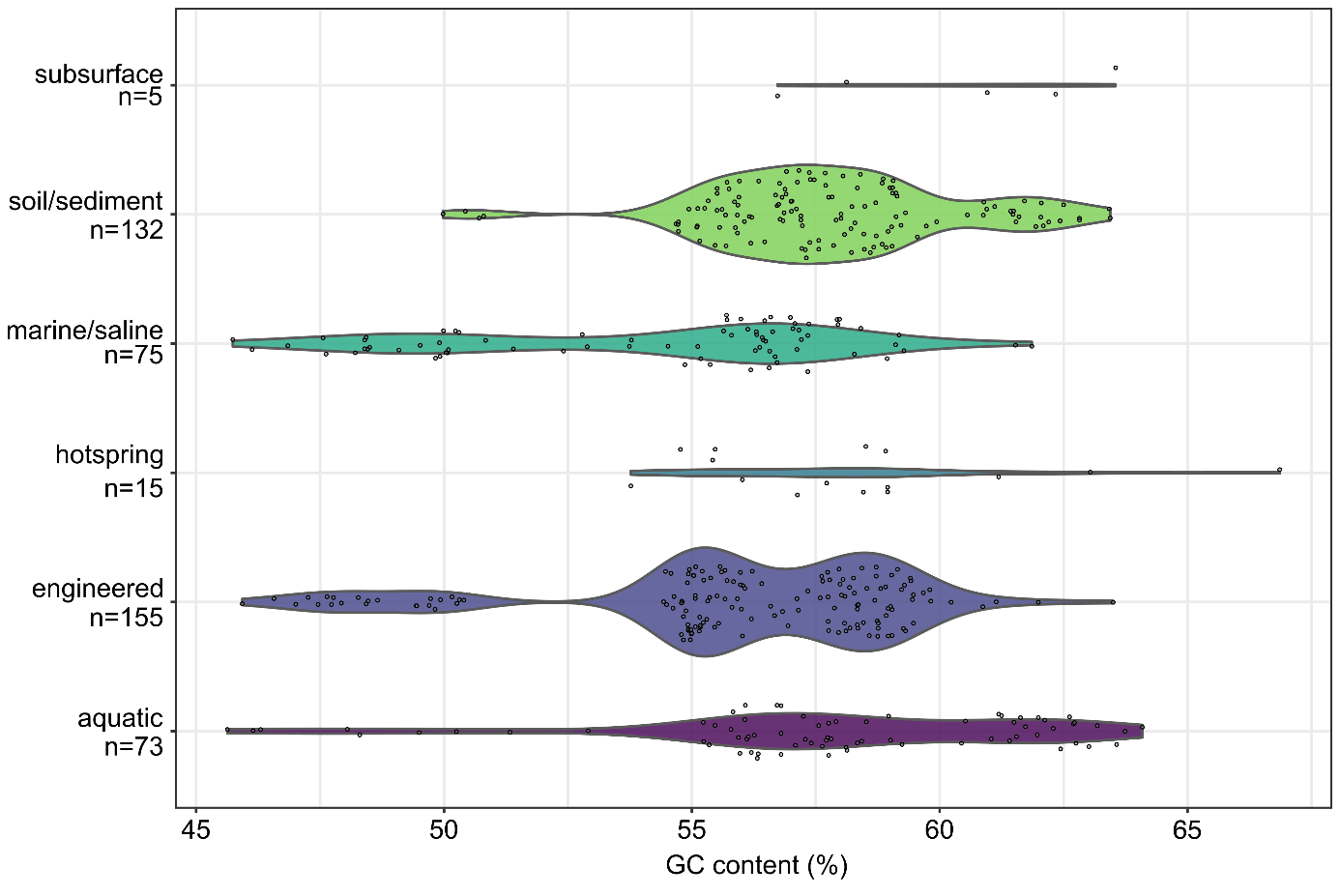


**Figure S17:** GC values (%) of uncultivated *Nitrospira* MAGs sorted by their habitats. Data are based on Kop et al. (2025). Habitat categories are according to Supplementary Table S3.

**Supplementary tables**

**Supplementary table S1:** 16S rRNA gene sequences obtained by amplicon sequencing and their taxonomical assignments.

**Supplementary table S2:** Accession number of 16S rRNA gene sequences and genome sequences used for phylogenetic tree calculation.

**Supplementary table S3:** GC content of *Nitrospira* MAGs.

**Supplementary table S4:** General characteristics of the draft genomes of *Ca.* N. neuquenensis E2OT and *Nitrospira* sp. Vd2.

**Supplementary table S5:** Pangenome analysis comparing *Ca.* Nitrospira neuquenensis E2OT, *Nitrospira* sp. KamNS4, *Nitrospira calida* Ns10, *Nitrospira* sp. Vd2, *Ca.* Nitrospira bockiana 47C and *Nitrospira tepida* using the pangenome tool of genoscope and following parameters: 50% amino acid identity and 80% alignment coverage.

**Supplementary table S6:** General characteristics of MAGs binned from the two metagenomes.

**Supplementary table S7:** GC content of cultivated *Nitrospira* species.

**Supplementary table S8:** Predicted coding sequences (CDS) and comparison of selected metabolic features of *Ca.* N. neuquenensis E2OT and *Nitrospira* sp. Vd2 with other moderately thermophilic *Nitrospira* species.

**Supplementary table S4:** General characteristics of the draft genomes of *Ca.* N. neuquenensis E2OT and *Nitrospira* sp. Vd2.

|  | **E2OT** | **Vd2** |
| --- | --- | --- |
| Genome length | 3,59 Mbp | 3,87 Mbp |
| GC content | 69.35 % | 60.63 % |
| Contigs | 39 | 42 |
| CDS (MAGE) | 3646 | 4090 |
| rRNA | 2 | 3 |
| tRNA (MAGE) | 43 | 44 |
| checkM genome completeness (MAGE) | 96.82 % | 95.85 % |
| checkM genome contamination (MAGE) | 2.27 % | 6.36 % |
| checkM2 genome completeness (MAGE) | 99.99 % | 99.95 % |
| checkM2 genome contamination (MAGE) | 0.8 % | 0.87 % |

**References**

Bayer, B., Saito, M.A., McIlvin, M.R., Lücker, S., Moran, D.M., Lankiewicz, T.S., Dupont, C.L., Santoro, A.E., 2021. Metabolic versatility of the nitrite-oxidizing bacterium *Nitrospira marina* and its proteomic response to oxygen-limited conditions. ISME J. 15, 1025–1039. https://doi.org/10.1038/s41396-020-00828-3

Bushnell, B., 2014. BBMap: A Fast, Accurate, Splice-Aware Aligner (No. LBNL-7065E). Lawrence Berkeley National Lab. (LBNL), Berkeley, CA (United States).

Capella-Gutiérrez, S., Silla-Martínez, J.M., Gabaldón, T., 2009. trimAl: a tool for automated alignment trimming in large-scale phylogenetic analyses. Bioinformatics 25, 1972–1973. https://doi.org/10.1093/bioinformatics/btp348

Caporaso, J.G., Kuczynski, J., Stombaugh, J., Bittinger, K., Bushman, F.D., Costello, E.K., Fierer, N., Peña, A.G., Goodrich, J.K., Gordon, J.I., Huttley, G.A., Kelley, S.T., Knights, D., Koenig, J.E., Ley, R.E., Lozupone, C.A., McDonald, D., Muegge, B.D., Pirrung, M., Reeder, J., Sevinsky, J.R., Turnbaugh, P.J., Walters, W.A., Widmann, J., Yatsunenko, T., Zaneveld, J., Knight, R., 2010. QIIME allows analysis of high-throughput community sequencing data. Nat. Methods 7, 335–336. https://doi.org/10.1038/nmeth.f.303

Caporaso, J.G., Lauber, C.L., Walters, W.A., Berg-Lyons, D., Lozupone, C.A., Turnbaugh, P.J., Fierer, N., Knight, R., 2011. Global patterns of 16S rRNA diversity at a depth of millions of sequences per sample. Proc. Natl. Acad. Sci. U. S. A. 108 Suppl 1, 4516–4522. https://doi.org/10.1073/pnas.1000080107

Chklovski, A., Parks, D.H., Woodcroft, B.J., Tyson, G.W., 2023. CheckM2: a rapid, scalable and accurate tool for assessing microbial genome quality using machine learning. Nat. Methods 20, 1203–1212. https://doi.org/10.1038/s41592-023-01940-w

Couvin, D., Bernheim, A., Toffano-Nioche, C., Touchon, M., Michalik, J., Néron, B., Rocha, E.P.C., Vergnaud, G., Gautheret, D., Pourcel, C., 2018. CRISPRCasFinder, an update of CRISRFinder, includes a portable version, enhanced performance and integrates search for Cas proteins. Nucleic Acids Res. 46, W246–W251. https://doi.org/10.1093/nar/gky425

Daims, H., Nielsen, J.L., Nielsen, P.H., Schleifer, K.-H., Wagner, M., 2001. In Situ Characterization of *Nitrospira* -Like Nitrite-Oxidizing Bacteria Active in Wastewater Treatment Plants. Appl. Environ. Microbiol. 67, 5273–5284. https://doi.org/10.1128/AEM.67.11.5273-5284.2001

Dowd, S.E., Sun, Y., Secor, P.R., Rhoads, D.D., Wolcott, B.M., James, G.A., Wolcott, R.D., 2008. Survey of bacterial diversity in chronic wounds using Pyrosequencing, DGGE, and full ribosome shotgun sequencing. BMC Microbiol. 8, 43. https://doi.org/10.1186/1471-2180-8-43

Edgar, R.C., 2004. MUSCLE: multiple sequence alignment with high accuracy and high throughput. Nucleic Acids Res. 32, 1792–1797. https://doi.org/10.1093/nar/gkh340

Edgar, R.C., Haas, B.J., Clemente, J.C., Quince, C., Knight, R., 2011. UCHIME improves sensitivity and speed of chimera detection. Bioinforma. Oxf. Engl. 27, 2194–2200. https://doi.org/10.1093/bioinformatics/btr381

Eren, A.M., Kiefl, E., Shaiber, A., Veseli, I., Miller, S.E., Schechter, M.S., Fink, I., Pan, J.N., Yousef, M., Fogarty, E.C., Trigodet, F., Watson, A.R., Esen, Ö.C., Moore, R.M., Clayssen, Q., Lee, M.D., Kivenson, V., Graham, E.D., Merrill, B.D., Karkman, A., Blankenberg, D., Eppley, J.M., Sjödin, A., Scott, J.J., Vázquez-Campos, X., McKay, L.J., McDaniel, E.A., Stevens, S.L.R., Anderson, R.E., Fuessel, J., Fernandez-Guerra, A., Maignien, L., Delmont, T.O., Willis, A.D., 2021. Community-led, integrated, reproducible multi-omics with anvi’o. Nat. Microbiol. 6, 3–6. https://doi.org/10.1038/s41564-020-00834-3

Eren, A.M., Maignien, L., Sul, W.J., Murphy, L.G., Grim, S.L., Morrison, H.G., Sogin, M.L., 2013. Oligotyping: differentiating between closely related microbial taxa using 16S rRNA gene data. Methods Ecol. Evol. 4, 1111–1119. https://doi.org/10.1111/2041-210X.12114

Eren, A.M., Morrison, H.G., Lescault, P.J., Reveillaud, J., Vineis, J.H., Sogin, M.L., 2015. Minimum entropy decomposition: unsupervised oligotyping for sensitive partitioning of high-throughput marker gene sequences. ISME J. 9, 968–979. https://doi.org/10.1038/ismej.2014.195

Ewels, P., Magnusson, M., Lundin, S., Käller, M., 2016. MultiQC: summarize analysis results for multiple tools and samples in a single report. Bioinforma. Oxf. Engl. 32, 3047–3048. https://doi.org/10.1093/bioinformatics/btw354

Herlemann, D.P.R., Labrenz, M., Jürgens, K., Bertilsson, S., Waniek, J.J., Andersson, A.F., 2011. Transitions in bacterial communities along the 2000 km salinity gradient of the Baltic Sea. ISME J. 5, 1571–1579. https://doi.org/10.1038/ismej.2011.41

Hoang, D.T., Chernomor, O., Von Haeseler, A., Minh, B.Q., Vinh, L.S., 2018. UFBoot2: Improving the Ultrafast Bootstrap Approximation. Mol. Biol. Evol. 35, 518–522. https://doi.org/10.1093/molbev/msx281

Kalyaanamoorthy, S., Minh, B.Q., Wong, T.K.F., Von Haeseler, A., Jermiin, L.S., 2017. ModelFinder: fast model selection for accurate phylogenetic estimates. Nat. Methods 14, 587–589. https://doi.org/10.1038/nmeth.4285

Kang, D.D., Li, F., Kirton, E., Thomas, A., Egan, R., An, H., Wang, Z., 2019. MetaBAT 2: an adaptive binning algorithm for robust and efficient genome reconstruction from metagenome assemblies. PeerJ 7, e7359. https://doi.org/10.7717/peerj.7359

Katoh, K., 2002. MAFFT: a novel method for rapid multiple sequence alignment based on fast Fourier transform. Nucleic Acids Res. 30, 3059–3066. https://doi.org/10.1093/nar/gkf436

Kim, D., Park, S., Chun, J., 2021. Introducing EzAAI: a pipeline for high throughput calculations of prokaryotic average amino acid identity. J. Microbiol. 59, 476–480. https://doi.org/10.1007/s12275-021-1154-0

Kisand, V., Cuadros, R., Wikner, J., 2002. Phylogeny of Culturable Estuarine Bacteria Catabolizing Riverine Organic Matter in the Northern Baltic Sea. Appl. Environ. Microbiol. 68, 379–388. https://doi.org/10.1128/AEM.68.1.379-388.2002

Koch, H., Galushko, A., Albertsen, M., Schintlmeister, A., Gruber-Dorninger, C., Lücker, S., Pelletier, E., Le Paslier, D., Spieck, E., Richter, A., Nielsen, P.H., Wagner, M., Daims, H., 2014. Growth of nitrite-oxidizing bacteria by aerobic hydrogen oxidation. Science 345, 1052–1054. https://doi.org/10.1126/science.1256985

Koch, H., Lücker, S., Albertsen, M., Kitzinger, K., Herbold, C., Spieck, E., Nielsen, P.H., Wagner, M., Daims, H., 2015. Expanded metabolic versatility of ubiquitous nitrite-oxidizing bacteria from the genus *Nitrospira*. Proc. Natl. Acad. Sci. 112, 11371–11376. https://doi.org/10.1073/pnas.1506533112

Kop, L.F.M., Koch, H., Speth, D., Lüke, C., Spieck, E., S M Jetten, M., Daims, H., Lücker, S., 2025. Comparative genome analysis reveals broad phylogenetic and functional diversity within the order Nitrospirales. ISME J. wraf151. https://doi.org/10.1093/ismejo/wraf151

Lebedeva, E.V., Off, S., Zumbrägel, S., Kruse, M., Shagzhina, A., Lücker, S., Maixner, F., Lipski, A., Daims, H., Spieck, E., 2011. Isolation and characterization of a moderately thermophilic nitrite-oxidizing bacterium from a geothermal spring: Moderately thermophilic Nitrospira-cultures from hot springs. FEMS Microbiol. Ecol. 75, 195–204. https://doi.org/10.1111/j.1574-6941.2010.01006.x

Letunic, I., Bork, P., 2007. Interactive Tree Of Life (iTOL): an online tool for phylogenetic tree display and annotation. Bioinforma. Oxf. Engl. 23, 127–128. https://doi.org/10.1093/bioinformatics/btl529

Li, H., 2013. Aligning sequence reads, clone sequences and assembly contigs with BWA-MEM. https://doi.org/10.48550/arXiv.1303.3997

Nurk, S., Meleshko, D., Korobeynikov, A., Pevzner, P.A., 2017. metaSPAdes: a new versatile metagenomic assembler. Genome Res. 27, 824–834. https://doi.org/10.1101/gr.213959.116

Pedersen, T., 2025. ggforce: Accelerating “ggplot2” [WWW Document]. URL https://thomasp85.r-universe.dev/ggforce (accessed 10.20.25).

Price, M.N., Dehal, P.S., Arkin, A.P., 2010. FastTree 2 – Approximately Maximum-Likelihood Trees for Large Alignments. PLOS ONE 5, e9490. https://doi.org/10.1371/journal.pone.0009490

Pritchard, L., Glover, R.H., Humphris, S., Elphinstone, J.G., Toth, I.K., 2016. Genomics and taxonomy in diagnostics for food security: soft-rotting enterobacterial plant pathogens. Anal. Methods 8, 12–24. https://doi.org/10.1039/C5AY02550H

Prjibelski, A., Antipov, D., Meleshko, D., Lapidus, A., Korobeynikov, A., 2020. Using SPAdes De Novo Assembler. Curr. Protoc. Bioinforma. 70, e102. https://doi.org/10.1002/cpbi.102

R Core Team (2022) R A Language and Environment for Statistical Computing. R Foundation for Statistical Computing, Vienna. - References - Scientific Research Publishing [WWW Document], n.d. URL https://www.scirp.org/reference/referencespapers?referenceid=3456808 (accessed 10.22.25).

Rognes, T., Flouri, T., Nichols, B., Quince, C., Mahé, F., 2016. VSEARCH: a versatile open source tool for metagenomics. PeerJ 4, e2584. https://doi.org/10.7717/peerj.2584

Shen, W., Sipos, B., Zhao, L., 2024. SeqKit2: A Swiss army knife for sequence and alignment processing. iMeta 3, e191. https://doi.org/10.1002/imt2.191

Speth, D.R., Pullen, N., Aroney, S.T.N., Coltman, B.L., Osvatic, J.T., Woodcroft, B.J., Rattei, T., Wagner, M., 2025. GlobDB: A comprehensive species-dereplicated microbial genome resource. https://doi.org/10.48550/arXiv.2506.11896

Turner, S., Pryer, K.M., Miao, V.P.W., Palmer, J.D., 1999. Investigating Deep Phylogenetic Relationships among Cyanobacteria and Plastids by Small Subunit rRNA Sequence Analysis1. J. Eukaryot. Microbiol. 46, 327–338. https://doi.org/10.1111/j.1550-7408.1999.tb04612.x

Vallenet, D., Calteau, A., Dubois, M., Amours, P., Bazin, A., Beuvin, M., Burlot, L., Bussell, X., Fouteau, S., Gautreau, G., Lajus, A., Langlois, J., Planel, R., Roche, D., Rollin, J., Rouy, Z., Sabatet, V., Médigue, C., 2020. MicroScope: an integrated platform for the annotation and exploration of microbial gene functions through genomic, pangenomic and metabolic comparative analysis. Nucleic Acids Res. 48, D579–D589. https://doi.org/10.1093/nar/gkz926

Wickham, H., 2016. Data Analysis, in: Wickham, H. (Ed.), Ggplot2: Elegant Graphics for Data Analysis. Springer International Publishing, Cham, pp. 189–201. https://doi.org/10.1007/978-3-319-24277-4_9

Wong, T.K.F., Ly-Trong, N., Ren, H., Banos, H., Roger, A.J., Susko, E., Bielow, C., De Maio, N., Goldman, N., Hahn, M.W., Huttley, G., Lanfear, R., Minh, B.Q. (2025) IQ-TREE 3: Phylogenomic Inference Software using Complex Evolutionary Models. https://doi.org/10.32942/X2P62N
