# Supplementary material for: Cultivation‐Based Detection of a Novel High‐GC Nitrospira Derived From the Argentinian Copahue Volcano Area
Source: Environ Microbiol. 2026 Apr 3;28(4):e70290. doi: 10.1111/1462-2920.70290 (PMC13049253; doi:10.1111/1462-2920.70290)
Supplement: Supplementary file 2 — Figure S13: Phylogenomic classification of Ca. N. neuquenensis E2OT and Nitrospira sp. Vd2 based on the concatenated alignment of 71 conserved bacterial marker proteins. The genomes of Ca. N. neuquenensis E2OT and Nitrospira sp. Vd2 are labelled in bold. Names of Nitrospirales genomes included in GlobDB R226 and the prefix “Candidatus” was omitted. Black circles indicate bootstrap support of 100%. [file EMI-28-e70290-s002.pdf]

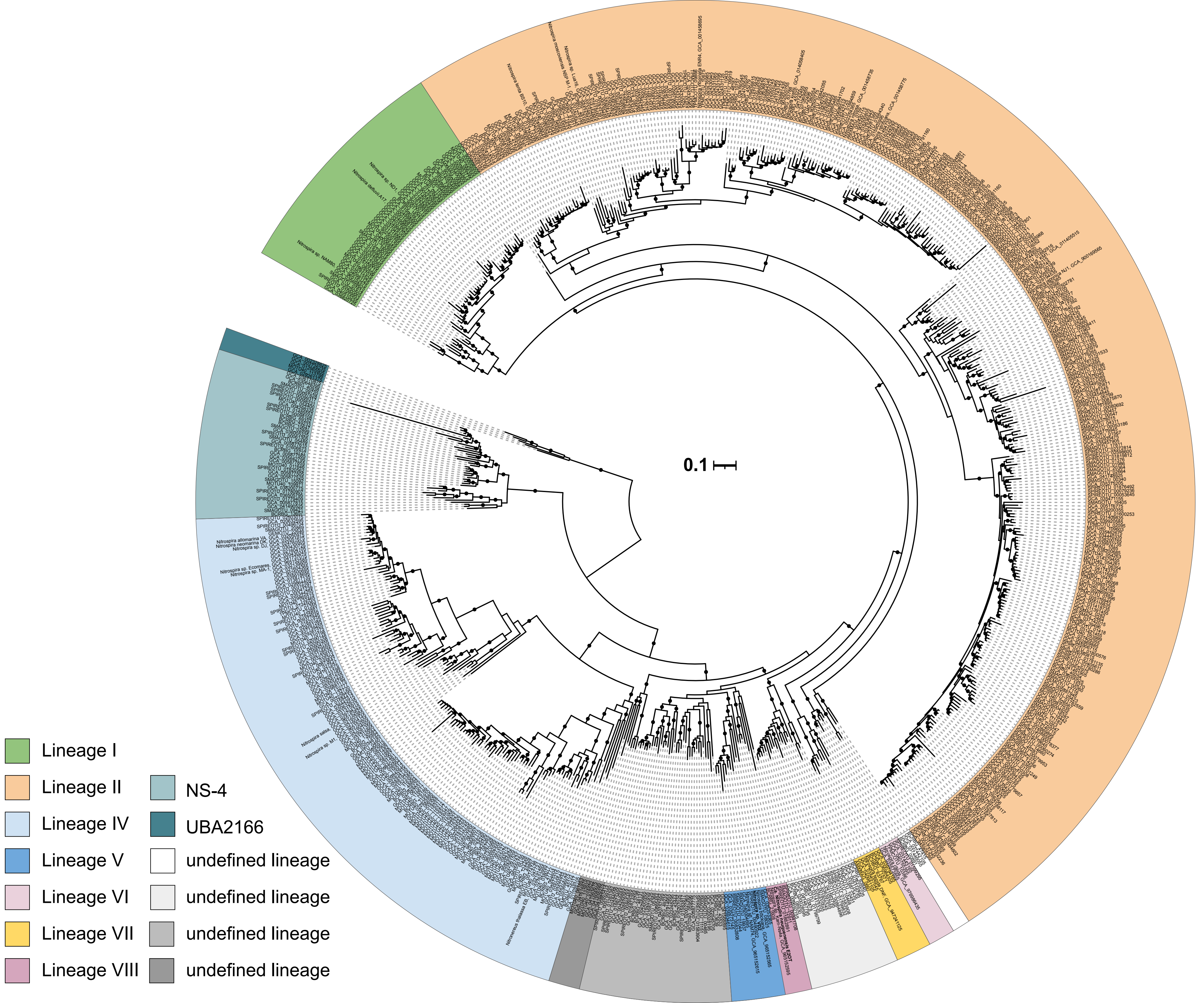
0.1
Lineage I
Lineage II
Lineage IV
Lineage V
Lineage VI
Lineage VII
Lineage VIII
NS-4
UBA2166
undefined lineage
undefined lineage
undefined lineage
undefined lineage
